# State of the Art of Invasive Group A Streptococcus Infection in Children: A Scoping Review of the Literature with a Focus on Predictors of Invasive Infection

**DOI:** 10.3390/children10091472

**Published:** 2023-08-29

**Authors:** Francesco Mariani, Carolina Gentili, Valentina Pulcinelli, Laura Martino, Piero Valentini, Danilo Buonsenso

**Affiliations:** 1 Department of Woman and Child Health and Public Health, Fondazione Policlinico Universitario A. Gemelli IRCCS, 00168 Rome, Italy; francesco.mariani.100292@gmail.com (F.M.); carolina.gentili01@gmail.com (C.G.); valentina.pulcinelli01@icatt.it (V.P.); martino.laura96@gmail.com (L.M.); piero.valentini@unicatt.it (P.V.); 2 Centro di Salute Globale, Università Cattolica del Sacro Cuore, 00168 Rome, Italy

**Keywords:** group A Streptococcus, GAS, invasive group A Streptococcus, iGAS, IVIG, clindamycin

## Abstract

Currently, it remains unclear why some children develop invasive group A Streptococcus (iGAS) and how to manage this condition. Therefore, to explore available works in the literature, we performed a scoping review aiming to analyze the current literature on clinical presentation of different illnesses outcomes of iGAS, with a specific focus on predictors of invasive infection, including an assessment of the prodromal stages of the disease and the possible presence of previous non-invasive GAS infections in children that later developed iGAS. Methods: We conducted a systematic search on PubMed and SCOPUS of all pediatric studies reporting iGAS cases, following the Preferred Reporting Items for Systematic reviews and Meta-Analyses extension for Scoping Reviews (PRISMA-ScR) checklist. For those studies in which multivariable analysis investigating iGAS risk factors was performed, a second review was performed and reported in detail. Results: A total of 209 studies were included. Five studies investigated risk factors for iGAS, the most relevant being varicella infection, chronic underlying illness, presence of the speC gene in GAS strains, acetaminophen and ibuprofen use, children nonwhite, living in low-income households, exposure to varicella at home, persistent high fever, having more than one other child in the home, and new use of NSAIDs. Although we observed a progressive increase in the number of papers published on this topic, no trials investigating the benefits of clindamycin or intravenous immunoglobulins were found and low-to-middle-income countries were found to be poorly represented in the current literature. Conclusions: Our scoping review highlights important gaps regarding several aspects of iGAS in children, including prodromic presentation and optimal treatment strategies. There is also little representation of low–middle-income countries. The current literature does not allow the performance of systematic reviews or meta-analyses, but this work should inform healthcare professionals, policy makers, and funding agencies on which studies to prioritize on this topic.

## 1. Introduction

*Streptococcus pyogenes* (group A Streptococcus, GAS) can cause a broad spectrum of infections, ranging from minor illnesses such as pharyngitis and superficial skin infections to severe and invasive diseases, including pneumonia, meningitis, sepsis, streptococcal toxic shock syndrome, and necrotizing fasciitis [1]. An invasive disease is defined as the isolation of GAS from a normally sterile site of the body and it occurs when bacteria spread throughout the bloodstream, the cerebrospinal fluid, the lungs, and soft tissue [1]. A critical virulence factor is the M protein [2,3]. Invasive group A Streptococcus infection (iGAS) is a life-threatening condition, with high case fatality rates and high morbidity. In fact, invasive disease can lead to several long-term sequelae, being an important cause of premature disability in pediatric populations [1].

Surveillance is essential to establish the real burden of this disease in children. According to the CDC’s Active Bacterial Core surveillance reports, overall iGAS incidence increased every year from 2012 to 2019; in 2020, especially during the first months of the pandemic period, the incidence of invasive disease saw a historical drop and the change was greatest for children aged 5 to 17 years [4]. Unexpectedly, the preliminary data from the surveillance reports of 2022 showed a monthly increase in the incidence of iGAS infection in children from September to November, thus leading the CDC to issue a health advisory [5]. Data from the first months of 2023 confirmed a higher incidence of these infections, compared to pre-pandemic levels. Different European countries reported to the ECDC an increase in iGAS disease in children aged less than 10 years from September 2022, with Ireland, France, and the UK reporting several deaths, too [6]. In the Netherlands, in 2022, a more than twofold increase in the number of iGAS infections compared to pre-pandemic reports was observed, with a higher increase in children under 5 years of age [7]. Otherwise, the UK experienced a surge in the number of invasive infections in children younger than 15 years [8]. However, the ongoing investigations have not yet found a new strain or increased antibiotic resistance, so the WHO has assessed the risk for the general population posed by iGAS infections as low [9]. Nevertheless, the report of higher than usual numbers of cases of iGAS has alarmed both the public and clinicians, with consequences for routine care. Higher rates of antibiotic prescriptions have been reported [10]. As a possible consequence, during these months of iGAS case number surges, a shortage of amoxicillin has been reported in most European countries [10].

One of the reasons that may have induced such public opinion is parents’ fear of GAS infection, and theoretical attempts for early diagnosis and treatment are goals of preventing non-invasive GAS infection from evolving into iGAS. However, so far, little is known about the natural history of iGAS and whether an invasive infection is the consequence of a non-recognized GAS infection or colonization. So far, we know that an increase in the incidence of iGAS is related to the high circulation of respiratory viruses, especially Respiratory Syncytial Virus (RSV) and seasonal influenza [11,12] and chickenpox can be a facilitating factor for the development of iGAS. Conversely, what is less known is whether it is possible to prevent the development of iGAS by early recognition and treatment of GAS infection. This last observation has direct implication on routine care because if iGAS suddenly begins as a sudden invasive infection without a previous phase of non-invasive GAS infection/colonization, there is no need for the public and healthcare professionals to be exaggeratedly scared of iGAS and, therefore, antibiotic use may be reduced. Conversely, if the literature suggests that iGAS develops after a non-treated possible or certain GAS infection, this suggests that better strategies are needed to achieve the goal of preventing the development of iGAS. 

For these reasons, we performed a scoping review aiming to analyze the current literature on clinical presentation of different localizations and the main outcomes of iGAS, with a specific focus on predictors of invasive infection, including an assessment of the prodromic stages of the disease and the possible presence of previous non-invasive GAS infections in children that later developed iGAS. Our team opted for a scoping review because it is currently unknown if there is any evidence that early treatment of non-complicated GAS infections can prevent iGAS or if iGAS is the consequence of an undiagnosed non-complicated GAS infection or if iGAS should be treated with intravenous immunoglobulins (IVIG) and clindamycin (or one of them) or not. Therefore, we decided to explore what types of paper are currently available as well as where and when available studies have been performed, aiming to inform researchers if systematic reviews and meta-analyses on this topic are feasible or not and to inform what studies are needed to fill current gaps.

## 2. Materials and Methods

### 2.1. Search Strategy and Selection Criteria for Included Studies

Our review aims to study a very broad topic to provide the scientific community with a picture of the current literature available in this regard. For this reason, a scoping review, conducted in line with a previously published protocol [13] and well-defined guidelines, seemed to us the best methodological choice. For reporting, we decided to follow the indication of the Preferred Reporting Items for Systematic reviews and Meta-Analyses extension for Scoping Reviews (PRISMA-ScR) checklist [14].

A single author (FM) developed a search strategy, subsequently discussed and approved by all the team, based on a combination of the following terms: “pediatric”, “iGAS (and its possible clinical manifestations)”, and “group A *Streptococcus pyogenes*”. We considered children and pediatric patients younger than 18 years. The search strategy for PubMed is available in the extended data section of the protocol. The terms used for this search were adapted for use with other bibliographic databases thanks to the Polyglot Function of Systematic Review Accelerator. PubMed and SCOPUS were the two databases screened and only articles written in English were included. We did not apply any data restrictions and included studies published until 31 January 2023. PubMed and SCOPUS searches were performed on the same day.

The main review question is “which are the predictors of clinical iGAS”?

This review also assess the following sub-questions:

1. Which are the most frequently reported localizations of different types of iGAS?
2. Which outcomes are reported in the literature about the different types of iGAS (pneumonia, meningitis, sepsis, and abscesses)?

This review includes studies performed on children and adolescents (younger than 18 years) with a confirmed diagnosis of iGAS defined as a laboratory isolation of GAS from any normal sterile site or isolation of GAS from a non-sterile site in patients with necrotizing fasciitis or streptococcal toxic shock syndrome. We include children diagnosed with pneumonia, sepsis, abscesses, or meningitis, due to GAS invasion.

One author screened for possible duplicates. Following the indication of the inclusion criteria, two reviewers (CG and VP) independently screened titles and abstracts and subsequently full texts to select works that could be included in the review. In case of a discrepancy between the two (we had 482 conflicts), a third reviewer (DB) decided whether to include the works.

### 2.2. Data Extraction

An initial data extraction was performed by a single reviewer (CG for half the included papers and VP for the other half) who compiled an initial Excel sheet in which the following information was collected: year of publication, type of study, region of origin, either retrospective or prospective study, either monocentric or multicentric study, if a multivariable analysis was performed or not, number of centers included, sample size, enrollment start and end date, length of participants’ follow-up, study eligibility criteria, age of patients, number of female patients, if analysis of comorbidities was performed or not, number of patients with comorbidities, if antibiotics were given, if intravenous immunoglobulins were given, if clindamycin was given, number of patients who died because of the disease, number of patients who died for other reasons, number of patients who survived with sequelae, and what type of sequelae were developed. 

Considering the high number of studies included, we decided to perform a second data extraction including only papers in which multivariable analysis investigating iGAS risk factors was performed. For those articles, the following information was collected and presented in a single table: number of patients with invasive infection, inclusion criteria, previous or starting symptoms or conditions which could lead to iGAS, associated factors detected through univariable or multivariable analysis, Streptococcal infection previously detected and not treated, previous therapy, and death from Streptococcal disease or sequelae due to the invasive infection. 

A critical appraisal was not pursued due to its limited relevance within the scope of a scoping review, postponing this objective to any future systematic reviews.

## 3. Results

**Figure 1:**
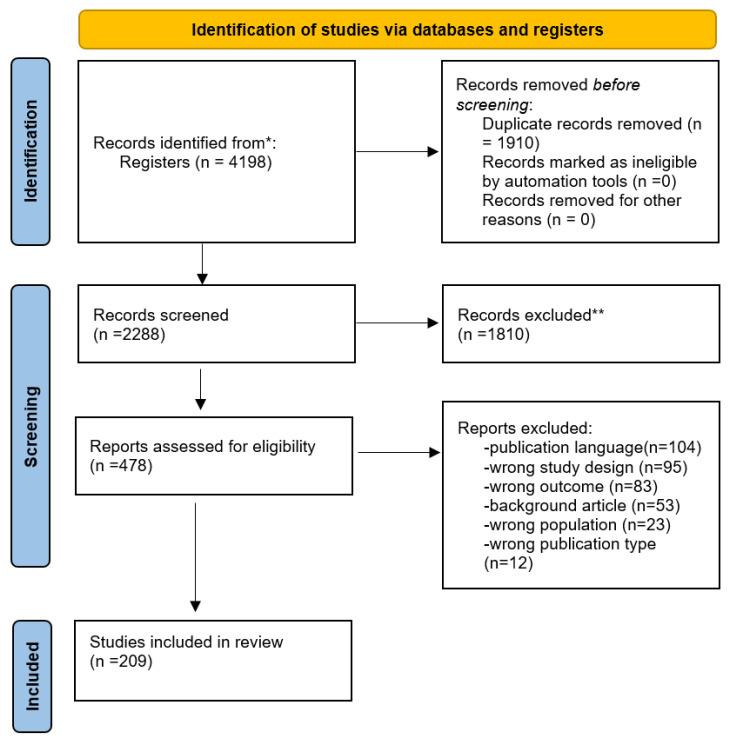
Study flowchart. *, ** not fulfilling study criteria.

Two-hundred and nine studies were included in our scoping review (Figure 1) and the general characteristics of the studies included are reported in Table 1. The full list of studies included in the scoping review is available in the Supplementary Materials.

Included papers described iGAS cases characterized by the following clinical localizations: CNS localizations (thirty-six studies), streptococcal toxic shock syndrome (thirty-five studies), skin (twenty-five studies), sepsis (twenty-three studies), cardiac (eight studies), gastrointestinal (six studies), pneumonia (six studies), osteomyelitis (five studies), kidneys (one study), and a variety of clinical iGAS localizations in fifty-five studies.

**Figure 2:**
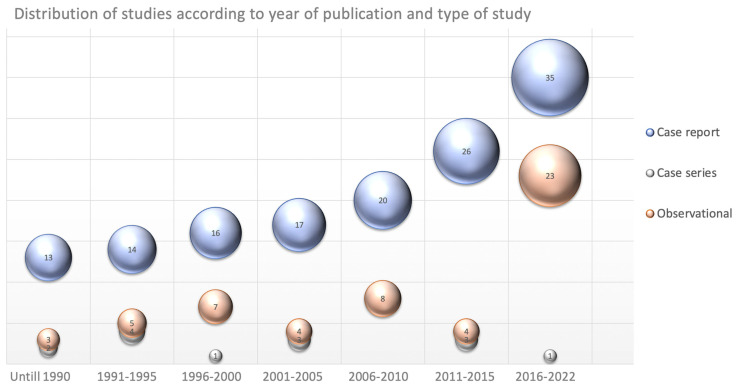
Distribution of studies according to type of study and year of publication.

It is possible to observe a progressive incrementation in the number of papers published on this topic over the years with most of the observational studies published in the last seven years. Figure 2 shows the distribution of studies according to type of study and year of publication.

Most of the papers (141, 67.5%), were case reports, 14 (6.7%) case series, and 54 (25.8%) observational studies. Eighty-seven studies (41.6%) were performed in North America, 60 (28.7%) in Europe, 47 (22.5%) in Asia, and the remaining 15 (7.2%) in Africa or Oceania or Central/South America. In only five studies, a multivariable analysis investigating factors associated with iGAS was performed. The cumulative number of patients in all the studies was 3068 (1222 females, 39.8%) with a mean age of 59.9 (±50.04) months and 1145 patients (37.3%) presented at least one comorbidity.

The most frequently reported therapies were clindamycin (for 594 patients, 19.4%), an antibiotic different from clindamycin (for 1667 patients, 54.3%), and immunoglobulins (for 196 patients, 6.4%). Not all the studies reported data about therapy.

Data about outcomes were collected too, pooled from all studies included, which included overall 3068 patients. Between the different studies, two-hundred and seven patients (6.7%) died from the disease, fifteen children (0.5%) died from other causes, and 169 children (5.5%) presented sequelae (Table 2).

In Table 3, the main characteristics of the five studies performing multivariable analysis are summarized [11,12,15,16,17]. None of these studies signaled a positive case of GAS detected before the onset of iGAS. According to the multivariable analysis, the risk factors reported were varicella infection, chronic underlying illness, chickenpox associated with the diagnosis of necrotizing fasciitis, presence of the speC gene in the GAS strain, acetaminophen and ibuprofen use, children nonwhite, living in low-income households, exposition to varicella at home, persistent high fever, having more than one other child in the home, and new use of NSAIDs.

## 4. Discussion

In this scoping review, we analyzed the existing literature, providing information on the risk factors, localizations, treatments, and outcomes of children with iGAS. In particular, our focus was to address if studies explored risk factors for iGAS, including common symptoms like pharyngitis/otitis and whether previous GAS infections and whether antibiotics were associated with the development of iGAS. Unfortunately, we found only five well-designed studies that could provide multivariate analyses on risk factors associated with iGAS, and no studies provided information about previous infection/colonization with GAS in children later diagnosed with iGAS, nor was information available about any antibiotic treatment of a potential prodromal phase of iGAS. In addition, no randomized controlled trials were found that explored the role of IVIG, in association or not in association with clindamycin, on the outcomes of iGAS. Our study, therefore, highlights important gaps in current knowledge which is reflected in the uncertainty of medical doctors about what to do to prevent iGAS infection. To our knowledge, this is the first systematic mapping, through a standardized scoping review approach based on PRISMA guidelines, of all available works in the literature on risk factors and treatments used for iGAS, including details about types of study. In particular, the strongest risk factors highlighted in the studies with multivariable analysis were varicella infection (OR, 6.2; 95% CI, 1.7–22.4) in the Minodier study [15]; association with the diagnosis of necrotizing fasciitis (RR, 4.5 (1.0–20)) in the Laupland study [12]; exposure to varicella (OR 6.4 (2.6–16)) in the Lesko study [17]); presence of the speC gene in GAS strains (OR, 4.0; 95% CI, 1.2–13.9) [15]; acetaminophen and ibuprofen use (according to Lesko [17], acetaminophen only use (OR, 0.94 (0.34–2.6)), ibuprofen only use (OR, 2.5 (0.58–11)), and both (acetaminophen and ibuprofen) (OR 5.6 (1.2–25)); according to Factor [11], (NSAIDs) OR, 10.64 (2.08–54.61)); children nonwhite; living in low-income households; exposure to varicella at home; persistent high fever; having more than one other child in the home; and new use of nonsteroidal anti-inflammatory drugs (NSAIDs) [11,12]. These data are mostly in line with previous publications in the literature describing that the presence of one other child living in the same house, varicella zoster virus infection, and the use of nonsteroidal anti-inflammatory drugs have been reported in the literature as risk factors for iGAS [11,12]. Of the five studies performing multivariate analyses and including a total of 487 children, details about the diagnosis of pharyngitis, otitis, or sinusitis before iGAS were reported only for 13, 4, and 2 children, respectively, and it was not clear for any of them if they were tested or not for GAS. This means that, in 2023, we are still unaware if a diagnosis of iGAS is preceded by a non-invasive infection (or colonization) with GAS and, as a consequence, it is completely unknown if low thresholds for testing and treating GAS may have any effect on reducing iGAS incidence, even in periods of high numbers of cases, as may happen in cold seasons with high circulation of respiratory viruses. As such, at the moment, clinicians should not test every child with mild symptoms or non-consistent symptoms for GAS nor routinely offer empiric treatment with penicillin or beta-lactams in an attempt to prevent iGAS. Similarly, such information should be transferred to the public in order to avoid the exaggerated fear of iGAS noticed during the 2022–23 winter season in Europe, which may have worse longer-term implications such as antibiotic shortages, development of drug resistance, and an overflow of patients in emergency departments. With the limited current state of knowledge, it is plausible that indiscriminate GAS testing and treatment may have a minimal effect in preventing iGAS infections.

To better understand if children with iGAS have, in the days or weeks before the disease, a possible GAS infection/colonization that might have been diagnosed and treated to prevent iGAS, new prospective studies are highly needed. In this regard, a new European pediatric prospective study (PEGASUS) is currently implementing an observational study on pediatric invasive group A streptococcal disease, with the aim of describing the incidence, risk factors, clinical phenotypes, microbiology and resistance, treatment, and outcomes of iGAS in children across Europe (https://www.pegasus-study.eu/, accessed on 20 July 2023).

Although our scoping review highlights that no studies investigated what happened in the weeks before the diagnosis of iGAS, the reinforcement that chickenpox is a strong risk factor in several studies may have public health implications. For example, some countries like United Kingdom do not routinely suggest chickenpox vaccination for children [18] for several reasons, including the usual self-limiting nature of the illness and the possible faster decline in induced immunity compared with natural immunity, which in turn can predispose zoster and other VZV complications in the elderly population [19]. However, considering the high burden of iGAS in countries like the UK and its association with previous cases of chickenpox, such a choice may be reconsidered (if not, the public should at least be made aware of this association and possible indirect benefit of the vaccination) to provide a more comprehensive decision to parents [20,21].

In this scoping, we mapped the therapeutic strategies used to manage iGAS infection. Currently, most guidelines do not give strong indications about the use of clindamycin and/or IVIG, although most experts use them. Clindamycin is a frequent choice given its action of ribosome and the consequent ability to inhibit protein synthesis, therefore limiting the production of GAS toxins [22]. Similarly, IVIG can neutralize toxin activity [23]. However, none of the studies that used clindamycin or IVIG were randomized or randomized controlled trials. As such, even a systematic review or meta-analysis to reply to this question would not be enough, while a RCT to understand the role of clindamycin, with or without IVIG, is highly needed. Currently, the ability to link multinational referral centers should allow such a study design. Of note, a recent Japanese nationwide observational study evaluated the effectiveness of intravenous immunoglobulin therapy for invasive group A Streptococcus infection [24]. A total of 481 patients (median age, 65 years; female, 49.7%) were included in the analysis. The overall mortality rate was 31.0%. After adjusting for background factors, we found that IVIG treatment had no effect on in-hospital mortality (adjusted odds ratio (OR): 0.99, 95% confidence interval (CI): 0.93–1.04, *p* = 0.92). Similar results were obtained after propensity score matching (OR: 1.00, 95% CI: 0.62–1.61, *p* > 0.99). The authors concluded that IVIG administration had no survival benefit in adult iGAS patients.

An important finding of this paper is the underrepresentation of iGAS publications from low-to-middle-income countries (LMICs). Paradoxically, LMICs are the countries that still suffer the most from non-suppurative consequences of GAS infections, including rheumatic fever [25]. Therefore, it is plausible to expect that these countries also have a higher incidence of GAS and iGAS infections, particularly if we consider that, in our scoping review, being of nonwhite ethnicity was also a risk factor in developing iGAS. This represents an important gap and this information should be used by policy makers and funding agencies to facilitate multinational studies on the topic that also include LMICs.

All together, the findings of our scoping review, despite highlighting current gaps in iGAS knowledge, may still provide some recommendations to healthcare professionals. First, we were not able to find studies evaluating whether iGAS is the consequence of an unrecognized uncomplicated GAS infection and, therefore, indiscriminate use of antibiotics in any tonsillitis case is not appropriate. However, some strong risk factors are coherent in several studies, such as previous cases of chickenpox. Therefore, a possible strategy for next winter to limit iGAS cases, in light of the unexpectedly high peak cases of 2022–2023, would be to expand the access to varicella vaccinations in children in those countries not recommending it. In a survey performed in the Netherlands, varicella infection preceded all cases of necrotizing fasciitis [26]. In the Netherlands, varicella vaccination is not included in the national immunization program and the 2022 varicella epidemic in the Netherlands was higher than expected, leading the authors to speculate if varicella infections contributed to recent surges of iGAS [27]. In periods of high GAS circulation and notification of iGAS cases, like in the current post-pandemic era, a rethinking of the indirect benefits of varicella vaccination on iGAS development may be warranted. Also, two studies [11,17] found an association of NSAIDs, confirmed by an older review as well [28]. Although a causative link has not been proven, hypotheses for such an effect have been proposed, including the ability of NSAIDs to interrupt the negative feedback loop that limits production of tumor necrosis factor (TNF)-alpha or its possible role in masking the signs and symptoms of developing severe infection [29,30]. As such, healthcare professionals and public policy makers may consider providing balanced information campaigns on the use of over-the-counter medications for pain and fever to the public, particularly in periods of high GAS circulation and unusual increase in notification rates of iGAS cases.

Our scoping review has some limitations. We only included studies published in English. Although we also attempted a mixed approach linking a mapping of the literature with a synthesis of main data when feasible, we did not perform a meta-analyses and sensitivity data reported should be considered as approximate to provide a general perspective of what is known on the topic. Also, our search was limited to the two databases SCOPUS and Pubmed. Although this is a limitation, we still screened as many as 4198 papers, and two is the minimum required numbers of databases for systematic reviews, and we only had access to them in our institution. Last, the article extraction process was performed by two authors due to the extensive number of articles, an approach that carries the potential for bias, although the two reviewers were trained on data extraction by the PI (DB) and the coordinator of the search strategy (FM).

## 5. Conclusions

In conclusion, our scoping review highlights important gaps on several aspects of iGAS in children, including prodromic presentation and optimal treatment strategies. No pediatric trials were found, and only a small number of studies have been performed in LMICs, where iGAS may have a higher incidence. Only four studies were prospective. Our findings should inform researchers, policy makers and funding agencies on which study designs should be prioritized on this topic, focusing efforts and funds on those studies that may help us in responding the most important questions on this topic: researchers have to better understand how we can recognize, and treat, early GAS infections to prevent the development of iGAS and clarify if the use of clindamycin, with or without IVIG, improves outcomes of iGAS.

## Figures and Tables

**Table 1:** Study characteristics.

	Number of Studies (N = 209)
**Type of study**	
RCT	0
NRCT	0
Observational	54
Case report	141
Case series	14
**Study design**	
Retrospective	205
Prospective	4
**Number of centers**	
Monocentric	177
Multicentric	32
**Region**	
Europe	60
Africa	6
North America	87
Central or South America	2
Asia	47
Oceania	7
**Multivariable analysis for iGAS risk factors performed**	5
**Information about comorbidities collected**	115

**Table 2:** Population cumulative characteristics.

	Patients (N = 3068)
Female	1222
Age (months)	
Mean (SD)	59.9 (±50.04)
Comorbidity	1145
Clindamycin in addition to other antibiotics	594
Intravenous immunoglobulin	196
Death from disease	207
Death from other causes	15
Alive with sequelae	169

**Table 3:** Characteristics of the studies with a regression analysis. * Both the analyses were controlled for sex and race; “new use of NSAIDs” (nonsteroidal anti-inflammatory drugs) indicates that a case patient started using NSAIDs in the 2 weeks before illness was diagnosed or that a control participant started using NSAIDs in the 2 weeks before the interview.

Author	Number of iGAS	Inclusion Criteria	Previous or Starting Symptoms or Conditions (Number of Patients)	Associated Factors Detected (Univariate Analysis)	Associated Factors Detected (Multivariable Analysis)	GAS Previously Detected and Not Treated	Therapy before iGAS Insurgence	Death for Disease	Sequelae (Number of Patients)
Minodier P, 2009 [15]	68	-Skin and soft tissue infections -Cardiopulmonary GAS -GAS Nervous system -Sepsis -Other iGAS	-Varicella within a month before iGAS (17)	-Gender female; OR, 0.72 (0.25–2.13) -Mean age ± SD; OR, 0.99 (0.98–1.01) -Recent varicella; OR, 7.5 (2.2–25.6) -Mean temperature ± SD; OR, 1.4 (0.8–2.5) -Mean delay symptoms ± SD; OR, 1.4 (0.9–2.03) -STSS; OR, 12.0 (2.1–6.7) -WBC; OR, 1.0 (1.0–1.0) -Polynuclear cells; OR, 1.0 (1.0–1.0) -Lymphocytes; OR, 1.0 (1.0–1.0) -PLT; OR, 1.0 (1.0–1.0) -CRP; OR, 0.99 (0.8–1.0) -GAS positive BC; OR, 0.08 (0.2–0.3) -emm 1 type; OR, 0.4 (0.1–1.5) -Virulence genes speA (OR, 0.4 (0.1–1.5)); speC (OR, 4.7 (1.5–14.7)); ssa (OR, 1.1 (0.3–4.2)); smeZ-1 (OR, 0.4 (0.1–1.5)); sic OR, 0.5 (0.1–1.8)	**-Varicella infection (OR, 6.2; 95% CI, 1.7–22.4)** **-Presence of *speC* gene in GAS strain (OR, 4.0; 95% CI, 1.2–13.9)**	Not specified	Not specified	3	Not specified
Stefanie Gauguet, 2015 [16]	86	-Sepsis -STSS -Skin and soft tissue	-Varicella (15) -Pharyngitis (13) -Other skin lesions (7) -Otitis media (4) -Sinusitis (2) -Central venous catheter infection (2) -Surgical wound infection (1)	-Comorbid illness; OR, 1.01 (0.35–2.87) -Immunosuppression; OR, 0.38 (0.08–1.82)	-Bacteriemia without a source. Adjusted OR, 0.08 (0.01–0.67)	Not specified	Not specified	2	Amputation (8)
Kevin B. Laupland, 2000 [12]	243	-Skin and soft tissue -Cardiopulmonary iGAS -GAS Nervous system -Sepsis -Osteomyelitis iGAS	-Varicella (31) -Asthma (12) -Malignancy (11) -Congenital cardiac disease (4) -Biliary atresia (3) -Juvenile rheumatoid arthritis (1) -Dermatomyositis (1) -Renal transplant (1) -Seizure disorder (1) -Ependymoma of brainstem (1) -Quadriplegia (1) -Cerebral palsy (1) -Multiple congenital anomalies (1)	-Leukemia; RR, 44 (30–56) -Asthma; RR, 0.74 (0.42–1.33) -Antecedent chickenpox associated with diagnosis of necrotizing fasciitis; RR, 6.3 (1.8–22.3)	**-Chronic underlying illness; RR, 11 (2.4–45)** **-Chickenpox associated with the diagnosis of necrotizing fasciitis (for** **the subset of children with soft tissue infection); RR, 4.5 (1.0–20)**	Not specified	Not specified	3	0
Samuel M. Lesko, 2001 [17]	52	-iGAS -Skin and soft tissue infection	-Varicella (52)	-Acetaminophen only use; OR, 0.96 (0.43–2.2) -Ibuprofen only use; OR, 1.5 (0.44–5.1) -Both (acetaminophen and ibuprofen); OR, 5.0 (1.6–16)	-Acetaminophen only use; OR, 0.94 (0.34–2.6) -Ibuprofen only use; OR, 2.5 (0.58–11) **-Both (acetaminophen and ibuprofen); OR, 5.6 (1.2–25)** **-Children nonwhite; OR, 3.8, (1.4–11)** **-Children living in low-income households; OR, 5.1 (1.7–15)** **-Exposed to varicella at home; OR, 6.4 (2.6–16)** **-Persistent high fever; OR, 9.6 (2.8–33).** -Use of ibuprofen before the development of signs or symptoms of this complication; OR, 1.3 (0.33–5.3)	Not specified	-Acetaminophen only, for Varicella symptoms (19) -Ibuprofen only (5) -Acetaminophen and ibuprofen (13)	0	0
Factor SH, 2005 [11]	38	iGAS	-VZV infection (3 patients) -HIV infection (1 patient)	* -Having a primary caretaker who smokes; OR, 2.71 (1.02–7.21) -Presence of >1 other child in the home; OR, 5.76 (1.95–16.96) -New use of NSAIDs; OR, 3.15 (1.07–9.29) -More rooms in the home; OR, 0.81 (0.66–0.99) -Higher level of parental education; OR, 0.69 (0.51–0.91) -A household member with a runny nose (rhinitis) in the past 2 weeks; OR, 0.25 (0.08–0.80)	* -Having >1 other child in the home; OR, 16.85 (3.9–72.84) **-//new use of nonsteroidal anti-inflammatory drugs (NSAIDs); OR, 10.64 (2.08–54.61)** -More rooms in the home; OR, 0.67 (0.51–0.88) -Having a household member with a runny nose in the past 2 weeks; OR, 0.09 (0.01–0.4)	Not specified	-NSAIDs (9 patients) -Steroids (1 patient)	2	Not specified

## Data Availability

Dataset available upon request to the corresponding author.

## Supplementary Materials

The following supporting information can be downloaded at: https://www.mdpi.com/article/10.3390/children10091472/s1.
