# Supplementary material for: State of the Art of Invasive Group A Streptococcus Infection in Children: A Scoping Review of the Literature with a Focus on Predictors of Invasive Infection
Source: Children (Basel). 2023 Aug 29;10(9):1472. doi: 10.3390/children10091472 (PMC10528266; doi:10.3390/children10091472)
Supplement: Supplementary file 1 [file children-10-01472-s001.zip › children-2532118-supplementary.pdf]

SUPPLEMENTARY MATERIAL: LIST OF STUDIES INCLUDED IN THE SCOPING REVIEW

1)A 7-year-old boy with acute onset of breathing difficulty

Salas AA. A 7-year-old boy with acute onset of breathing difficulty. Pediatr Emerg Care. 2010 Feb;26(2):149-51. doi: 10.1097/PEC.0b013e3181d0a04e. PMID: 20145509.

2)A Case of Meningitis Caused by Streptococcus pyogenes in a Child with Ventriculoperitoneal Shunt and Ommaya Reservoir
Karaaslan, Ayse, et al. "A Case of Meningitis Caused by Streptococcus pyogenes in a Child with Ventriculoperitoneal Shunt and Ommaya Reservoir/Ventriküloperitoneal Santi ve Ommaya Rezervuari Olan Çocukta Streptokokkus pyogenes Menenjiti." *Cocuk Enfeksiyon Dergisi* 7.1 (2013): 35.

3)A case report of hemolytic streptococcal gangrene in the danger triangle of the face with thrombocytopenia and hepatitis
Jia XL, Pathak JL, Tong JF, Su JM. A case report of hemolytic streptococcal gangrene in the danger triangle of the face with thrombocytopenia and hepatitis. BMC Pediatr. 2018 Jun 22;18(1):198. doi: 10.1186/s12887-018-1177-9. PMID: 29933752; PMCID: PMC6013964.

4)A cluster of paediatric invasive group a streptococcal and chicken pox infections
Ó Maoldomhnaigh C, Butler K, Gavin P. A Cluster of Paediatric Invasive Group A Streptococcal and Chicken Pox Infections. Ir Med J. 2018 Mar 14;111(3):718. PMID: 33959990.

5)A multi-center clinical investigation on invasive Streptococcus pyogenes infection in China,2010-2017
Hua CZ, Yu H, Xu HM, Yang LH, Lin AW, Lyu Q, Lu HP, Xu ZW, Gao W, Chen XJ, Wang CQ, Jing CM. A multi-center clinical investigation on invasive Streptococcus pyogenes infection in China, 2010-2017. BMC Pediatr. 2019 Jun 5;19(1):181. doi: 10.1186/s12887-019-1536-1. PMID: 31167650; PMCID: PMC6549372.

6) A rare association between group A Streptococcus purpura fulminans and diffuse alveolar hemorrhage in a young girl: A case report
Cousin E, Gaillot T, Ozanne B, Ryckewaert A, Dessard S, Tirel O, Fausser JL. A rare association between group A Streptococcus purpura fulminans and diffuse alveolar hemorrhage in a young girl: A case report. Arch Pediatr. 2021 Aug;28(6):488-490. doi: 10.1016/j.arcped.2021.04.012. Epub 2021 Jul 8. PMID: 34246502.

7) A rare case of infant sepsis due to the emm-89 genotype of Group A Streptococcus within a community-acquired cluster.
Pignanelli S, Brusa S, Pulcrano G, Catania MR, Cocchi E, Lanari M. A rare case of infant sepsis due to the emm-89 genotype of Group A Streptococcus within a community-acquired cluster. New Microbiol. 2015 Oct;38(4):589-92. Epub 2015 Oct 20. PMID: 26485019.

8) A rare case of osteomyelitis of the clavicle in a child due to Group A streptococcal infection
Ahmed MI, Nadeem M, Bandi S. A rare case of osteomyelitis of the clavicle in a child due to Group A streptococcal infection. BMJ Case Rep. 2019 Apr 11;12(4):e227090. doi: 10.1136/bcr-2018-227090. PMID: 30975773; PMCID: PMC6506016.

9)A serious and rare complication following varicella infection; streptococcal toxic shock syndrome-like case report
Aslan, Mustafa Törehan, et al. "A serious and rare complication following varicella infection; streptococcal toxic shock syndrome-like case report." *Cocuk Enfeksiyon Dergisi* 11.4 (2017): E162-E165.

10)Acute necrotising fasciitis due to streptococcal infection in a newborn infant

Nutman J, Henig E, Wilunsky E, Reisner SH. Acute necrotising fasciitis due to streptococcal infection in a newborn infant. Arch Dis Child. 1979 Aug;54(8):637-9. doi: 10.1136/adc.54.8.637. PMID: 389176; PMCID: PMC1545788.

11)Apparent increase in the incidence of invasive group A beta-hemolytic streptococcal disease in children
Givner LB, Abramson JS, Wasilauskas B. Apparent increase in the incidence of invasive group A beta-hemolytic streptococcal disease in children. J Pediatr. 1991 Mar;118(3):341-6. doi: 10.1016/s0022-3476(05)82144-4. PMID: 1999773.

12)Bilateral peritonsillar abscess: A case report and pertinent literature review
AlAwadh I, Aldrees T, AlQaryan S, Alharethy S, AlShehri H. Bilateral peritonsillar abscess: A case report and pertinent literature review. Int J Surg Case Rep. 2017;36:34-37. doi: 10.1016/j.ijscr.2017.04.028. Epub 2017 May 15. PMID: 28531866; PMCID: PMC5440272.

13)Brain abscess caused by streptococcus pyogenes in a previously healthy child
Dehority W, Uchiyama S, Khosravi A, Nizet V. Brain abscess caused by Streptococcus pyogenes in a previously healthy child. J Clin Microbiol. 2006 Dec;44(12):4613-5. doi: 10.1128/JCM.01724-06. Epub 2006 Oct 25. PMID: 17065264; PMCID: PMC1698393.

14) Bullous impetigo: a rare presentation in fulminant streptococcal toxic shock syndrome.
Lin JJ, Wu CT, Hsia SH, Chiu CH. Bullous impetigo: a rare presentation in fulminant streptococcal toxic shock syndrome. Pediatr Emerg Care. 2007 May;23(5):318-20. doi: 10.1097/01.pec.0000270166.62991.8c. PMID: 17505275.

15)Case report: Streptococcal toxic shock syndrome presenting as septic thrombophlebitis in a child with varicella
Cohen-Abbo A, Harper MB. Case report: streptococcal toxic shock syndrome presenting as septic thrombophlebitis in a child with varicella. Pediatr Infect Dis J. 1993 Dec;12(12):1033-4. PMID: 8108215.

16) Cellulitis and sepsis caused by group a streptococcus in Saudi neonate: Case report
Sobaih, Badr Hasan, and Adrian Hadid. "Cellulitis and Sepsis Caused by Group A Streptococcus in Saudi Neonate: Case Report." Kuwait Medical Journal 48.3 (2016): 253-254.

17) Cellulitis-adenitis in a neonate with group a streptococcal sepsis
Huber BM. Cellulitis-adenitis in a neonate with group a streptococcal sepsis. Klin Padiatr. 2014 Apr;226(2):82-3. doi: 10.1055/s-0033-1363255. Epub 2014 Mar 14. PMID: 24633980.

18) Characteristics of Intracranial Group A Streptococcal Infections in US Children, 1997-2014.
Link-Gelles R, Toews KA, Schaffner W, Edwards KM, Wright C, Beall B, Barnes B, Jewell B, Harrison LH, Kirley PD, Lorentzson L, Aragon D, Petit S, Bareta J, Spina NL, Cieslak PR, Van Beneden C. Characteristics of Intracranial Group A Streptococcal Infections in US Children, 1997-2014. J Pediatric Infect Dis Soc. 2020 Feb 28;9(1):30-35. doi: 10.1093/jpids/piy108. PMID: 30462264; PMCID: PMC8931553.

19) Clinical and epidemiologic features of invasive group A streptococcal infections in children
Mulla ZD. Clinical and epidemiologic features of invasive group A streptococcal infections in children. Pediatr Int. 2007 Jun;49(3):355-8. doi: 10.1111/j.1442-200X.2007.02378.x. PMID: 17532835.

20) Clinical and Laboratory Features of Invasive Group A Streptococcal Infections: 8 Years Experience
Şahin A, Yüksel NC, Karadağ Öncel E, Kara Aksay A, Yılmaz N, Yılmaz Çiftdoğan D. Clinical and Laboratory Features of Invasive Group A Streptococcal Infections: 8 Years Experience. Turk Arch Pediatr. 2022 Jan;57(1):75-80. doi: 10.5152/TurkArchPediatr.2022.21221. PMID: 35110082; PMCID: PMC8867505.

21) Clinical and microbiologic characteristics of group A streptococcal necrotizing fasciitis in children.
Minodier P, Bidet P, Rallu F, Tapiero B, Bingen E, Ovetchkine P. Clinical and microbiologic characteristics of group A streptococcal necrotizing fasciitis in children. Pediatr Infect Dis J. 2009 Jun;28(6):541-3. doi: 10.1097/inf.0b013e318195bb10. PMID: 19504739.

22) Clinical characteristics of children with group A streptococcal toxic shock syndrome admitted to pediatric intensive care units.
Rodríguez-Nuñez A, Dosil-Gallardo S, Jordan I; ad hoc Streptococcal Toxic Shock Syndrome collaborative group of Spanish Society of Pediatric Intensive Care. Clinical characteristics of children with group A streptococcal toxic shock syndrome admitted to pediatric intensive care units. Eur J Pediatr. 2011 May;170(5):639-44. doi: 10.1007/s00431-010-1337-x. Epub 2010 Oct 28. PMID: 20981441.

23) Clinical Description and Outcomes of Australian Children With Invasive Group A Streptococcal Disease.
Thielemans E, Oliver J, McMinn A, Baker C, Britton PN, Clark J, Marshall H, Blyth CC, Francis J, Buttery J, Smeesters PR, Crawford N, Steer AC. Clinical Description and Outcomes of Australian Children With Invasive Group A Streptococcal Disease. Pediatr Infect Dis J. 2020 May;39(5):379-384. doi: 10.1097/INF.0000000000002596. PMID: 32091492.

24) Diagnosis and treatment of streptococcal toxic shock syndrome in the pediatric intensive care unit: a case report.
Carvalho HT, Fioretto JR, Ribeiro CF, Laraia IO, Carpi MF. Diagnosis and treatment of streptococcal toxic shock syndrome in the pediatric intensive care unit: a case report. Rev Bras Ter Intensiva. 2019 Oct-Dec;31(4):586-591. doi: 10.5935/0103-507X.20190068. PMID: 31967236; PMCID: PMC7009001.

25) Emergence of invasive group A Streptococcal disease among young children
Novotny W, Faden H, Mosovich L. Emergence of invasive group A streptococcal disease among young children. Clin Pediatr (Phila). 1992 Oct;31(10):596-601. doi: 10.1177/000992289203101004. PMID: 1395366.

26) Endogenous endophthalmitis as a severe complication following a Streptococcus pyogenes infection.
Martinod M, Bost-Bru C, Mortamet G. Endogenous endophthalmitis as a severe complication following a Streptococcus pyogenes infection. Arch Pediatr. 2020 Nov;27(8):506-508. doi: 10.1016/j.arcped.2020.08.012. Epub 2020 Oct 1. PMID: 33011024.

27) Fatal group a streptococcal toxic shock-like syndrome in a child with varicella: Report of the first well documented case with detection of the genetic sequences that code for exotoxins spe A and B, in São Paulo, Brazil
Sztajnbok J, Lovgren M, Brandileone MC, Marotto PC, Talbot JA, Seguro AC. Fatal group A Streptococcal toxic shock-like syndrome in a child with varicella: report of the first well documented case with detection of the genetic sequences that code for exotoxins spe A and B, in São Paulo, Brazil. Rev Inst Med Trop Sao Paulo. 1999 Jan-Feb;41(1):63-5. doi: 10.1590/s0036-46651999000100011. PMID: 10436672.

28) Fatal necrotizing pneumonia caused by group A streptococcus
Cengiz AB, Kanra G, Caĝlar M, Kara A, Güçer S, Ince T. Fatal necrotizing pneumonia caused by group A streptococcus. J Paediatr Child Health. 2004 Jan-Feb;40(1-2):69-71. doi: 10.1111/j.1440-1754.2004.00296.x. PMID: 14718011.

29) Fulminant liver failure in a child with invasive group A streptococcal infection
Biesel-Desthieux MN, Tissières P, Belli DC, Le Coultre C, Gervaix A, Masserey Spicher V. Fulminant liver failure in a child with invasive group A streptococcal infection. Eur J Pediatr. 2003 Apr;162(4):245-7. doi: 10.1007/s00431-002-1137-z. Epub 2003 Feb 6. PMID: 12647197.

30) Group A beta-hemolytic streptococcal bacteremia in a patient with sickle cell anemia on penicillin prophylaxis.
LeBlanc W, Salah H, Khakoo Y. Group A beta-hemolytic streptococcal bacteremia in a patient with sickle cell anemia on penicillin prophylaxis. J Natl Med Assoc. 1995 May;87(5):347-8. PMID: 7783241; PMCID: PMC2607793.

31) Group A beta-hemolytic streptococcal bacteremia in children
Yagupsky P, Giladi Y. Group A beta-hemolytic streptococcal bacteremia in children. Pediatr Infect Dis J. 1987 Nov;6(11):1036-9. PMID: 3320930.

32) Group A beta-hemolytic streptococcal bacteremia.
Abuhammour W, Hasan RA, Unuvar E. Group A beta-hemolytic streptococcal bacteremia. Indian J Pediatr. 2004 Oct;71(10):915-9. doi: 10.1007/BF02830836. PMID: 15531835.

33) Group A beta-hemolytic streptococcal colitis with secondary bacteremia.
Arthur C, Linam LE, Linam WM. Group A beta-hemolytic streptococcal colitis with secondary bacteremia. Pediatr Infect Dis J. 2012 Oct;31(10):1093-5. doi: 10.1097/INF.0b013e31826318c6. PMID: 22683673.

34) Group A beta-hemolytic streptococcal epiglottitis as a complication of varicella infection.
Belfer RA. Group A beta-hemolytic streptococcal epiglottitis as a complication of varicella infection. Pediatr Emerg Care. 1996 Jun;12(3):203-4. doi: 10.1097/00006565-199606000-00013. PMID: 8806145.

35) Group a beta-hemolytic streptococcal pneumonia: clinical course and complications of management.
Molteni RA. Group a beta-hemolytic streptococcal pneumonia: clinical course and complications of management. Am J Dis Child. 1977 Dec;131(12):1366-71. doi: 10.1001/archpedi.1977.02120250048008. PMID: 337794.

36) Group A beta-hemolytic streptococcal septicemia complicating infected hemangioma in children.
Yagupsky P, Giladi Y. Group A beta-hemolytic streptococcal septicemia complicating infected hemangioma in children. Pediatr Dermatol. 1987 May;4(1):24-6. doi: 10.1111/j.1525-1470.1987.tb00748.x. PMID: 3295826.

37) Group a beta-hemolytic streptococcal toxic shock-like syndrome
Begovac J, Marton E, Lisić M, Beus I, Bozinović D, Kuzmanović N. Group A beta-hemolytic streptococcal toxic shock-like syndrome. Pediatr Infect Dis J. 1990 May;9(5):369-70. doi: 10.1097/00006454-199005000-00013. PMID: 2352822.

38) Group A beta-hemolytic streptococci as a cause of bacteremia in children.
Wong VK, Wright HT Jr. Group A beta-hemolytic streptococci as a cause of bacteremia in children. Am J Dis Child. 1988 Aug;142(8):831-3. doi: 10.1001/archpedi.1988.02150080037016. PMID: 3293424.

39) Group A beta–hemolytic streptococcus and waterhouse–friderichsen syndrome
Gertner M, Rodriguez L, Barnett SH, Shah K. Group A beta-hemolytic Streptococcus and Waterhouse-Friderichsen syndrome. Pediatr Infect Dis J. 1992 Jul;11(7):595-6. doi: 10.1097/00006454-199207000-00019. PMID: 1528655.

40) Group a streptococcal bacteremia without a source is associated with less severe disease in children
Gauguet S, Ahmed AA, Zhou J, Pfoh ER, Ahnger-Pier KK, Harper MB, Ozonoff A, Wessels MR, Lee GM. Group A streptococcal bacteremia without a source is associated with less severe disease in children. Pediatr Infect Dis J. 2015 Apr;34(4):447-9. doi: 10.1097/INF.0000000000000587. PMID: 25319760; PMCID: PMC4357552.

SUPPLEMENTARY MATERIAL: LIST OF STUDIES INCLUDED IN THE SCOPING REVIEW

41) Group A Streptococcal Brain Abscess in the Pediatric Population: Case Series and Review of the Literature.
Capua T, Klivitsky A, Bilavsky E, Ashkenazi-Hoffnung L, Roth J, Constantini S, Grisaru-Soen G. Group A Streptococcal Brain Abscess in the Pediatric Population: Case Series and Review of the Literature. Pediatr Infect Dis J. 2018 Oct;37(10):967-970. doi: 10.1097/INF.0000000000001947. PMID: 29462105.

42) Group A streptococcal brain abscess: A case report and a review of the literature since 1988
Group A streptococcal brain abscess.
Hayashi A, Takano T, Suzuki A, Narumiya S. Group A streptococcal brain abscess: a case report and a review of the literature since 1988. Scand J Infect Dis. 2011 Jul;43(6-7):553-5. doi: 10.3109/00365548.2011.564648. Epub 2011 Mar 9. PMID: 21385096.

43)Group A streptococcal meningitis as a complication of an infected capillary haemangioma
Rezvani M, Yager JY, Hartfield DS. Group A streptococcal meningitis as a complication of an infected capillary haemangioma. Eur J Pediatr. 2004 Jan;163(1):19-21. doi: 10.1007/s00431-003-1334-4. Epub 2003 Nov 11. PMID: 14610671.

44) Group A streptococcal meningitis complicating varicella.
Gradon JD, Chopnick EK, Lutwick LI, Tepperberg J, Kahn M. Group A streptococcal meningitis complicating varicella. Pediatr Infect Dis J. 1991 Oct;10(10):786-7. PMID: 1945585.

45) Group a streptococcal meningitis in a pediatric patient
Fanella S, Embree J. Group A streptococcal meningitis in a pediatric patient. Can J Infect Dis Med Microbiol. 2008 Jul;19(4):306-8. doi: 10.1155/2008/594583. PMID: 19436512; PMCID: PMC2604778.

46) Group A streptococcal meningitis in a pediatric patient following cochlear implantation: report of the first case and review of the literature.
Pettersen G, Ovetchkine P, Tapiero B. Group A streptococcal meningitis in a pediatric patient following cochlear implantation: report of the first case and review of the literature. J Clin Microbiol. 2005 Nov;43(11):5816-8. doi: 10.1128/JCM.43.11.5816-5818.2005. PMID: 16272530; PMCID: PMC1287785.

47) Group A streptococcal meningitis in a previously healthy infant
Gupta, Puneet Kumar; Chauhan, Smriti; Guleria, Ramesh Chand; Kanga, Anil; Singh, Digvijay. Group A streptococcal meningitis in a previously healthy infant. journal-of-pediatric-infectious-diseases/jpi00357. Sun Jan 01 00:00:00 GMT 2012. Doi: 10.3233/JPI-120357.

48) Group a streptococcal meningitis without predisposing factors
Brown CC, Pickering LK, Baker CJ. Group A streptococcal meningitis without predisposing factors. South Med J. 1981 Aug;74(8):1029-30. doi: 10.1097/00007611-198108000-00049. PMID: 7022665.

49) Group A streptococcal meningitis: a case report.
Busetti M, Marchetti F, Croci E, L'erario I, Creti R, D'Agaro P. Group A streptococcal meningitis: a case report. New Microbiol. 2013 Oct;36(4):419-22. Epub 2013 Oct 1. PMID: 24177305.

50) Group A streptococcal meningitis: Case report and review of the literature
Perera N, Abulhoul L, Green MR, Swann RA. Group A streptococcal meningitis: case report and review of the literature. J Infect. 2005 Aug;51(2):E1-4. doi: 10.1016/j.jinf.2004.06.006. PMID: 16038740.

51) Group a streptococcal meningitis: Report of two cases
Moses AE, Beeri M, Engelhard D. Group A streptococcal meningitis: report of two cases. J Infect. 1998 Jan;36(1):116-8. doi: 10.1016/s0163-4453(98)93558-7. PMID: 9515681.

52) Group a streptococcal necrotizing fasciitis following varicella in children: Case reports and review

Wilson GJ, Talkington DF, Gruber W, Edwards K, Dermody TS. Group A streptococcal necrotizing fasciitis following varicella in children: case reports and review. Clin Infect Dis. 1995 May;20(5):1333-8. doi: 10.1093/clinids/20.5.1333. PMID: 7620020.

53) Group A streptococcal osteomyelitis: Severe presentation and course
Turner D, Einhorn M. Group A streptococcal osteomyelitis: severe presentation and course. Acta Paediatr. 2003;92(1):125-7. doi: 10.1111/j.1651-2227.2003.tb00484.x. PMID: 12650315.

54) Group A streptococcal pharyngitis associated with primary peritonitis
Sharp EA, Linn A, Zitelli BJ. Group A streptococcal pharyngitis associated with primary peritonitis. BMJ Case Rep. 2019 May 6;12(5):e229186. doi: 10.1136/bcr-2019-229186. PMID: 31061197; PMCID: PMC6510132.

55) Group A streptococcal primary peritonitis in a healthy girl
Chomton M, Emeriaud G, Bidet P, Rallu F, Ovetchkine P, Gaschignard J. Group A streptococcal primary peritonitis in a healthy girl. J Paediatr Child Health. 2017 Jun;53(6):615-616. doi: 10.1111/jpc.13584. PMID: 28573799.

56) Group A streptococcal septic vasculitis in a child with dengue hemorrhagic fever
Vitug MR, Dayrit JF, Oblepias MS, Honrado ER. Group A streptococcal septic vasculitis in a child with dengue hemorrhagic fever. Int J Dermatol. 2006 Dec;45(12):1458-61. doi: 10.1111/j.1365-4632.2006.03084.x. PMID: 17184265.

57) Group A streptococcal septicemia, meningitis and cerebral abscess: case report and literature review.
Paul SP, Jerwood S. Group A streptococcal septicemia, meningitis and cerebral abscess: case report and literature review. Turk J Pediatr. 2012 Mar-Apr;54(2):180-3. PMID: 22734307.

58) Group A streptococcal subdural empyema as a complication of varicella
Ulloa-Gutierrez R, Dobson S, Forbes J. Group A streptococcal subdural empyema as a complication of varicella. Pediatrics. 2005 Jan;115(1):e112-4. doi: 10.1542/peds.2004-1336. PMID: 15629962.

59) Group A streptococcal toxic shock syndrome and associated respiratory distress.
Friedman O, Cook SP. Group A streptococcal toxic shock syndrome and associated respiratory distress. Otolaryngol Head Neck Surg. 1999 Apr;120(4):566-9. doi: 10.1053/hn.1999.v120.a82447. PMID: 10187961.

60) Group a streptococcal tracheitis associated with toxic shock syndrome
Burns JA, Brown J, Ogle JW. Group A streptococcal tracheitis associated with toxic shock syndrome. Pediatr Infect Dis J. 1998 Oct;17(10):933-5. doi: 10.1097/00006454-199810000-00024. PMID: 9802646.

61) Group A Streptococcus and hepatitis - Streptococcal toxic shock syndrome
Kenny, Benjamin; Gupta, Sanjeev; Brumby, Jarrod; Burton, Joanna; Moloney, Susan E. Group A Streptococcus and hepatitis – streptococcal toxic shock syndrome. journal-of-pediatric-infectious-diseases/jpi00173. Thu Jan 01 00:00:00 GMT 2009.doi 10.3233/JPI-2009-0173

62) Group A Streptococcus bacteremia among infants: A study from tertiary health care center of North India
Singla, Nidhi, et al. "Group A Streptococcus bacteremia among infants: A study from tertiary health care center of North India." Annals of Tropical Medicine & Public Health 5.6 (2012).

63) Group A Streptococcus puerperal sepsis with invasive neonatal infection: A fatal case.

Siti Hafsyah MH, Ibrahim NR, Noraida R, Ismail AA, Anani Aila MZ, Hajissa K, Zeehaida M. Group A Streptococcus puerperal sepsis with invasive neonatal infection: A fatal case. Med J Malaysia. 2021 Sep;76(5):731-733. PMID: 34508383.

64) Group A β-hemolytic streptococcal hemorrhagic colitis complicated with pharyngitis and impetigo
Isozaki A, Matsubara K, Yui T, Kobayashi K, Kawano Y. Group A beta-hemolytic streptococcal hemorrhagic colitis complicated with pharyngitis and impetigo. J Infect Chemother. 2007 Dec;13(6):411-3. doi: 10.1007/s10156-007-0550-7. Epub 2007 Dec 25. PMID: 18095091.

65) Increased incidence and severity of Streptococcus pyogenes bacteremia in young children.
Moses AE, Ziv A, Harari M, Rahav G, Shapiro M, Englehard D. Increased incidence and severity of Streptococcus pyogenes bacteremia in young children. Pediatr Infect Dis J. 1995 Sep;14(9):767-70. doi: 10.1097/00006454-199509000-00007. PMID: 8559625.

66) Invasive disease due to group A beta-hemolytic streptococci: continued occurrence in children in North Carolina.
Givner LB. Invasive disease due to group A beta-hemolytic streptococci: continued occurrence in children in North Carolina. South Med J. 1998 Apr;91(4):333-7. doi: 10.1097/00007611-199804000-00003. PMID: 9563422.

67) Invasive group A streptococcal disease in children and association with varicella-zoster virus infection. Ontario Group A Streptococcal Study Group.
Laupland KB, Davies HD, Low DE, Schwartz B, Green K, McGeer A. Invasive group A streptococcal disease in children and association with varicella-zoster virus infection. Ontario Group A Streptococcal Study Group. Pediatrics. 2000 May;105(5):E60. doi: 10.1542/peds.105.5.e60. PMID: 10799624.

68) Invasive group A streptococcal infection after tonsillectomy
Timmers-Raaijmaakers BC, Wolfs TF, Jansen NJ, Bos AP, van Vught AJ. Invasive group A streptococcal infection after tonsillectomy. Pediatr Infect Dis J. 2003 Oct;22(10):929-31. doi: 10.1097/01.inf.0000091361.22838.77. PMID: 14579819.

69) Invasive group A streptococcal infection and nonsteroidal antiinflammatory drug use among children with primary varicella
Lesko SM, O'Brien KL, Schwartz B, Vezina R, Mitchell AA. Invasive group A streptococcal infection and nonsteroidal antiinflammatory drug use among children with primary varicella. Pediatrics. 2001 May;107(5):1108-15. doi: 10.1542/peds.107.5.1108. PMID: 11331694.

70) Invasive group A streptococcal infection in the Northern Territory, Australia: case report and review of the literature.
Middleton B, Morris P, Carapetis J. Invasive group A streptococcal infection in the Northern Territory, Australia: case report and review of the literature. J Paediatr Child Health. 2014 Nov;50(11):869-73. doi: 10.1111/jpc.12659. Epub 2014 Jun 23. PMID: 24957474.

71) Invasive group A streptococcal infections in children with varicella in Southern California.
Vugia DJ, Peterson CL, Meyers HB, Kim KS, Arrieta A, Schlievert PM, Kaplan EL, Werner SB. Invasive group A streptococcal infections in children with varicella in Southern California. Pediatr Infect Dis J. 1996 Feb;15(2):146-50. doi: 10.1097/00006454-199602000-00011. PMID: 8822288.

72) Invasive group A Streptococcus disease in Australian children: 2016 to 2018 - a descriptive cohort study.
Oliver J, Thielemans E, McMinn A, Baker C, Britton PN, Clark JE, Marshall HS, Blyth CC, Francis J, Buttery J, Steer AC, Crawford NW; PAEDS investigators. Invasive group A Streptococcus disease in Australian children: 2016 to 2018 - a descriptive cohort study. BMC Public Health. 2019 Dec 30;19(1):1750. doi:

10.1186/s12889-019-8085-2. Erratum in: BMC Public Health. 2021 May 3;21(1):855. PMID: 31888568; PMCID: PMC6937995.

73) Invasive Group A Streptococcus Infection among Children, Rural Kenya.
Seale AC, Davies MR, Anampiu K, Morpeth SC, Nyongesa S, Mwarumba S, Smeesters PR, Efstratiou A, Karugutu R, Mturi N, Williams TN, Scott JA, Kariuki S, Dougan G, Berkley JA. Invasive Group A Streptococcus Infection among Children, Rural Kenya. Emerg Infect Dis. 2016 Feb;22(2):224-32. doi: 10.3201/eid2202.151358. PMID: 26811918; PMCID: PMC4734542.

74) Invasive Group A Streptococcus Infection in Children in Central Israel in 2012-2019.
Canetti M, Carmi A, Paret G, Goldberg L, Adler A, Amit S, Rokney A, Ron M, Grisaru-Soen G. Invasive Group A Streptococcus Infection in Children in Central Israel in 2012-2019. Pediatr Infect Dis J. 2021 Jul 1;40(7):612-616. doi: 10.1097/INF.0000000000003087. PMID: 34097654.

75) Invasive group A streptococcus infection presenting as purulent pericarditis with multiple splenic abscesses: case report and literature review.
Pemira SM, Tolan RW Jr. Invasive group A streptococcus infection presenting as purulent pericarditis with multiple splenic abscesses: case report and literature review. Clin Pediatr (Phila). 2012 May;51(5):436-41. doi: 10.1177/0009922811430345. Epub 2011 Dec 8. PMID: 22157427.

76) Invasive group A Streptococcus resulting in sepsis and abdominal wall abscess after adenotonsillectomy.
Wilson PF, Wannemuehler TJ, Matt BH. Invasive group A Streptococcus resulting in sepsis and abdominal wall abscess after adenotonsillectomy. Laryngoscope. 2015 May;125(5):1230-2. doi: 10.1002/lary.24997. Epub 2014 Nov 1. PMID: 25363250.

77) Invasive Streptococcus pyogenes infection - A retrospective clinical analysis of 6 cases
Grunert, Maria; Malik, Michalina; Lewandowska, Kaja; Machura, Edyta. Invasive Streptococcus pyogenes infection - A retrospective clinical analysis of 6 cases. Paediatrics and Family Medicine. (2021): 57–63. DOI:10.15557/PiMR.2021.0009

78) Invasive Streptococcus pyogenes Infections in <3-Month-Old Infants in France: Clinical and Laboratory Features.
Germont Z, Bidet P, Plainvert C, Bonacorsi S, Poyart C, Biran V, Frérot A, Faye A, Basmaci R. Invasive *Streptococcus pyogenes* Infections in <3-Month-Old Infants in France: Clinical and Laboratory Features. Front Pediatr. 2020 May 6;8:204. doi: 10.3389/fped.2020.00204. PMID: 32435626; PMCID: PMC7217982.

79) Invasive Streptococcus pyogenes infections in children
Cimolai N, Trombley C, Adderley RJ, Tredwell SJ. Invasive Streptococcus pyogenes infections in children. Can J Public Health. 1992 May-Jun;83(3):230-3. PMID: 1525751.

80) Mandibular osteomyelitis after group A streptococcal septicaemia in a child.
Ilahi NT, Awal D, Mills C, Ayliffe P. Mandibular osteomyelitis after group A streptococcal septicaemia in a child. Br J Oral Maxillofac Surg. 2017 Apr;55(3):341-342. doi: 10.1016/j.bjoms.2016.08.020. Epub 2016 Sep 12. PMID: 27633906.

81) Manifestations of Toxic Shock Syndrome in Children, Columbus, Ohio, USA, 2010-2017(1).
Cook A, Janse S, Watson JR, Erdem G. Manifestations of Toxic Shock Syndrome in Children, Columbus, Ohio, USA, 2010-2017[1]. Emerg Infect Dis. 2020 Jun;26(6):1077-1083. doi: 10.3201/eid2606.190783. PMID: 32442091; PMCID: PMC7258457.

82) Meningitis caused by toxigenic group a beta-hemolytic streptococcus in a pediatric patient with acquired immunodeficiency syndrome
Marshall GS, Patel CC, Buck G. Meningitis caused by toxigenic group A beta-hemolytic Streptococcus in a pediatric patient with acquired immunodeficiency syndrome. Pediatr Infect Dis J. 1991 Apr;10(4):339-40. doi: 10.1097/00006454-199104000-00018. PMID: 2062634.

83) Meningitis in a girl with recurrent otitis media caused by Streptococcus pyogenes - Otitis media has to be treated appropriately
Steppberger K, Adams I, Deutscher J, Müller H, Kiess W. Meningitis in a girl with recurrent otitis media caused by Streptococcus pyogenes--otitis media has to be treated appropriately. Infection. 2001 Oct;29(5):286-8. doi: 10.1007/s15010-001-1065-2. PMID: 11688910.

84) Molecular Characterization of Streptococcus pyogenes Causing Invasive Disease in Pediatric Population in Spain A 12-year Study.
Sánchez-Encinales V, Ludwig G, Tamayo E, García-Arenzana JM, Muñoz-Almagro C, Montes M. Molecular Characterization of Streptococcus pyogenes Causing Invasive Disease in Pediatric Population in Spain A 12-year Study. Pediatr Infect Dis J. 2019 Dec;38(12):1168-1172. doi: 10.1097/INF.0000000000002471. PMID: 31738331.

85) Multifocal bacterial osteomyelitis after varicella infection: a rare but dreaded complication of chickenpox.
Gallach Sanchis D, Doñate Pérez F, Jiménez Ortega P. Multifocal bacterial osteomyelitis after varicella infection: a rare but dreaded complication of chickenpox. Musculoskelet Surg. 2014 Jun;98(1):61-5. doi: 10.1007/s12306-012-0185-4. Epub 2012 Jun 17. PMID: 22707015.

86) Risk factors for invasive group A streptococcal infections in children with varicella: A case-control study
Peterson CL, Vugia DJ, Meyers HB, Chao SM, Vogt J, Lanson J, Brunell PA, Kim KS, Mascola L. Risk factors for invasive group A streptococcal infections in children with varicella: a case-control study. Pediatr Infect Dis J. 1996 Feb;15(2):151-6. doi: 10.1097/00006454-199602000-00012. PMID: 8822289.

87) Orthopaedic manifestations of invasive group A streptococcal infections complicating primary varicella.
Mills WJ, Mosca VS, Nizet V. Orthopaedic manifestations of invasive group A streptococcal infections complicating primary varicella. J Pediatr Orthop. 1996 Jul-Aug;16(4):522-8. doi: 10.1097/00004694-199607000-00021. PMID: 8784712.

88) When should clinicians suspect group A streptococcus empyema in children? A multicentre case–control study in French tertiary care centres
Bellulo S, Sommet J, Lévy C, Gillet Y, Hees L, Lorrot M, Gras-Le-Guen C, Craiu I, Dubos F, Minodier P, Biscardi S, Dommergues MA, Béchet S, Bidet P, Alberti C, Cohen R, Faye A; French Pediatric Infectious Diseases Study Group (GPIP). When should clinicians suspect group A streptococcus empyema in children? A multicentre case-control study in French tertiary care centres. Arch Dis Child. 2016 Aug;101(8):731-5. doi: 10.1136/archdischild-2015-309831. Epub 2016 Apr 12. PMID: 27073159.

89) Two cases and a review of Streptococcus pyogenes endocarditis in children
Weidman DR, Al-Hashami H, Morris SK. Two cases and a review of Streptococcus pyogenes endocarditis in children. BMC Pediatr. 2014 Sep 10;14:227. doi: 10.1186/1471-2431-14-227. PMID: 25208720; PMCID: PMC4169802.

90) Sudden Unexpected Death in a Child With Varicella Caused by Necrotizing Fasciitis and Streptococcal Toxic Shock Syndrome

SUPPLEMENTARY MATERIAL: LIST OF STUDIES INCLUDED IN THE SCOPING REVIEW

Hidalgo-Carballal A, Suárez-Mier MP. Sudden unexpected death in a child with varicella caused by necrotizing fasciitis and streptococcal toxic shock syndrome. Am J Forensic Med Pathol. 2006 Mar;27(1):93-6. doi: 10.1097/01.paf.0000203152.62134.1f. PMID: 16501360.

91) Subpectoral abscess a rare group A beta-hemolytic Streptococcus infection
Abuelreish MA, Rathore MH. Subpectoral abscess a rare group A beta-hemolytic Streptococcus infection. Pediatr Infect Dis J. 2005 Dec;24(12):1121-2. doi: 10.1097/01.inf.0000190037.79533.34. PMID: 16371884.

92) Streptococcus pyogenes-purpura fulminans as an invasive form of group A streptococcal infection.
Okuzono S, Ishimura M, Kanno S, Sonoda M, Kaku N, Motomura Y, Nishio H, Oba U, Hanada M, Fukushi JI, Urata M, Kang D, Takada H, Ohga S. Streptococcus pyogenes-purpura fulminans as an invasive form of group A streptococcal infection. Ann Clin Microbiol Antimicrob. 2018 Jul 9;17(1):31. doi: 10.1186/s12941-018-0282-9. PMID: 29986727; PMCID: PMC6036671.

93) Streptococcus pyogenes meningitis: Report of a case and review of the literature
Berner R, Herdeg S, Gordjani N, Brandis M. Streptococcus pyogenes meningitis: report of a case and review of the literature. Eur J Pediatr. 2000 Jul;159(7):527-9. doi: 10.1007/s004310051325. PMID: 10923229.

94) Streptococcus pyogenes cervical intertrigo with secondary bacteremia
López-Corominas V, Yagüe F, Knöpfel N, Dueñas J, Gil J, Martín-Santiago A, Hervás JA. Streptococcus pyogenes cervical intertrigo with secondary bacteremia. Pediatr Dermatol. 2014 Mar-Apr;31(2):e71-2. doi: 10.1111/pde.12256. Epub 2014 Jan 23. PMID: 24456009.

95) Streptococcus pyogenes Associated Post-traumatic Brodie's Abscess of Cuboid: A Case Report and Review of Literature
Amit P, Maharajan K, Varma B. Streptococcus pyogenes Associated Post-traumatic Brodie's Abscess of Cuboid: A Case Report and Review of Literature. J Orthop Case Rep. 2015 Jul-Sep;5(3):84-6. doi: 10.13107/jocr.2250-0685.318. PMID: 27299080; PMCID: PMC4719413.

96) Streptococcus associated toxic shock
Torres-Martínez C, Mehta D, Butt A, Levin M. Streptococcus associated toxic shock. Arch Dis Child. 1992 Jan;67(1):126-30. doi: 10.1136/adc.67.1.126. PMID: 1739326; PMCID: PMC1793551.

97) Streptococcal toxic shock-like syndrome: case report and review of the literature
al Mazrou AM. Streptococcal toxic shock-like syndrome: case report and review of the literature. Ann Trop Paediatr. 1994;14(2):145-8. doi: 10.1080/02724936.1994.11747707. PMID: 7521630.

98) Streptococcal toxic shock syndrome in children
Floret D, Stamm D, Cochat P, Delmas P, Kohler W. Streptococcal toxic shock syndrome in children. Intensive Care Med. 1992;18(3):175-6. doi: 10.1007/BF01709243. PMID: 1644967.

99) Streptococcal Toxic Shock Syndrome in a Child With Venous Malformation
Yoshida T, Asakura Y, Miura S, Endo M, Akasaka M. Streptococcal Toxic Shock Syndrome in a Child With Venous Malformation. Cureus. 2022 Jan 10;14(1):e21096. doi: 10.7759/cureus.21096. PMID: 35165555; PMCID: PMC8829108.

100) Streptococcal Toxic Shock Syndrome
Krishna V, Sankaranarayan S, Sivaraman RP, Prabaharan K. Streptococcal Toxic Shock syndrome. Indian J Pediatr. 2014 Sep;81(9):946-8. doi: 10.1007/s12098-013-1272-8. Epub 2013 Dec 3. PMID: 24297338.

101) Streptococcal pharyngitis: An uncommon cause of subdural empyema

Walden JH, Hess B, Rigby M. Streptococcal pharyngitis: an uncommon cause of subdural empyema. BMJ Case Rep. 2015 Sep 18;2015:bcr2015211312. doi: 10.1136/bcr-2015-211312. PMID: 26385939; PMCID: PMC4577616.

102) Splenic abscess caused by group A β-haemolytic streptococcus
Chang KW, Chiu CH, Jaing TH, Wong HF. Splenic abscess caused by group A beta-haemolytic streptococcus. Acta Paediatr. 2003 Apr;92(4):510-1. doi: 10.1111/j.1651-2227.2003.tb00589.x. PMID: 12801124.

103) Similarities and Differences Between Staphylococcal and Streptococcal Toxic Shock Syndromes in Children: Results From a 30-Case Cohort.
Javouhey E, Bolze PA, Jamen C, Lina G, Badiou C, Poyart C, Portefaix A, Tristan A, Laurent F, Bes M, Vandenesch F, Gilletand Y, Dauwalder O. Similarities and Differences Between Staphylococcal and Streptococcal Toxic Shock Syndromes in Children: Results From a 30-Case Cohort. Front Pediatr. 2018 Nov 28;6:360. doi: 10.3389/fped.2018.00360. PMID: 30547021; PMCID: PMC6280580.

104) Severe group A streptococcal infections in a paediatric intensive care unit.
Lithgow A, Duke T, Steer A, Smeesters PR. Severe group A streptococcal infections in a paediatric intensive care unit. J Paediatr Child Health. 2014 Sep;50(9):687-92. doi: 10.1111/jpc.12601. Epub 2014 Jun 9. PMID: 24909187.

105) Sacroiliitis infected with group A streptococcus in a child presenting with confusion and combativeness.
Komatsu H, Nojiri H, Sogo T, Inui A, Sawa F, Fujisawa T. Sacroiliitis infected with group A streptococcus in a child presenting with confusion and combativeness. J Infect Chemother. 2009 Oct;15(5):328-30. doi: 10.1007/s10156-009-0709-5. Epub 2009 Oct 24. PMID: 19856073.

106) Renal Interstitial Invasion by Group A Streptococcus: A Rare Presentation.
Lomanta F, Upadhyayula S. Renal Interstitial Invasion by Group A *Streptococcus*: A Rare Presentation. Case Rep Infect Dis. 2022 May 9;2022:5881375. doi: 10.1155/2022/5881375. PMID: 35586457; PMCID: PMC9110243.

107) Recurrent invasive group A streptococcal infection with four-limb amputation in an immunocompetent child
Gazzaz N, Mailman T, Foster JR. Recurrent invasive group A streptococcal infection with four-limb amputation in an immunocompetent child. BMJ Case Rep. 2018 Jul 30;2018:bcr2018225292. doi: 10.1136/bcr-2018-225292. PMID: 30061131; PMCID: PMC6069930.

108) Rapidly progressing subperiosteal orbital abscess: an unexpected complication of a group-A streptococcal pharyngitis in a healthy young patient
Costantinides F, Luzzati R, Tognetto D, Bazzocchi G, Biasotto M, Tirelli GC. Rapidly progressing subperiosteal orbital abscess: an unexpected complication of a group-A streptococcal pharyngitis in a healthy young patient. Head Face Med. 2012 Oct 16;8:28. doi: 10.1186/1746-160X-8-28. PMID: 23067784; PMCID: PMC3517307.

109) Purpura fulminans caused by group A beta-hemolytic Streptococcus sepsis.
Dhodapkar K, Corbacioglu S, Chang MW, Karpatkin M, DiMichele D. Purpura fulminans caused by group A beta-hemolytic Streptococcus sepsis. J Pediatr. 2000 Oct;137(4):562-7. doi: 10.1067/mpd.2000.109926. PMID: 11035839.

110) Puerperal fever and neonatal pleural empyema and bacteremia caused by group A streptococcus
Lequier L, Vaudry WL. Puerperal fever and neonatal pleural empyema and bacteremia caused by group A streptococcus. Can J Infect Dis. 1998 May;9(3):185-8. doi: 10.1155/1998/470984. PMID: 22346542; PMCID: PMC3250907.

SUPPLEMENTARY MATERIAL: LIST OF STUDIES INCLUDED IN THE SCOPING REVIEW

111) Primary psoas abscess caused by group A streptococcus in a child: Case report with microbiologic findings.
Kamiya Y, Hasegawa T, Takegami Y, Horiba K, Ando S, Torii Y, Kidokoro H, Kato T, Natsume J, Kawada JI, Ito Y. Primary psoas abscess caused by group A streptococcus in a child: Case report with microbiologic findings. J Infect Chemother. 2016 Dec;22(12):811-814. doi: 10.1016/j.jiac.2016.06.011. Epub 2016 Sep 28. PMID: 27692341.

112) Primary group A streptococcal septic shock syndrome simulating perforated appendicitis in a previously healthy girl
Patel RV, Kumar H, More B, Rajimwale A. Primary group A streptococcal septic shock syndrome simulating perforated appendicitis in a previously healthy girl. BMJ Case Rep. 2013 May 4;2013:bcr2013009502. doi: 10.1136/bcr-2013-009502. PMID: 23645702; PMCID: PMC3670001.

113) Primary Group A Streptococcal Peritonitis in a Previously Healthy Female Teenage Patient
Haskett H, Delair S, Neemann K. Primary Group A Streptococcal Peritonitis in a Previously Healthy Female Teenage Patient. Glob Pediatr Health. 2020 Sep 16;7:2333794X20957647. doi: 10.1177/2333794X20957647. PMID: 32984445; PMCID: PMC7498967.

114) Perianal streptococcal cellulitis with penile involvement.
Duhra P, Ilchyshyn A. Perianal streptococcal cellulitis with penile involvement. Br J Dermatol. 1990 Dec;123(6):793-6. doi: 10.1111/j.1365-2133.1990.tb04199.x. PMID: 2265096.

115) Pediatric necrotizing soft tissue infection after elective surgery: A case report and literature review.
Gheuens L, Roggeman Q, Cortebeeck K, Leyman P, Bernaerts A, De Foer B, Van Leemput J, van Dinther J. Pediatric necrotizing soft tissue infection after elective surgery: A case report and literature review. Int J Pediatr Otorhinolaryngol. 2020 Nov;138:110195. doi: 10.1016/j.ijporl.2020.110195. Epub 2020 Jul 10. PMID: 32705989.

116) Pediatric invasive streptococcal infection in northern and eastern regions of Hokkaido, Japan from 2010 to 2012.
Sakata H. Pediatric invasive streptococcal infection in northern and eastern regions of Hokkaido, Japan from 2010 to 2012. Pediatr Int. 2014 Jun;56(3):360-3. doi: 10.1111/ped.12250. Epub 2014 Mar 10. PMID: 24274786.

117) Pattern of varicella and associated complications in children in United Arab Emirates: 5-Year descriptive study
Uduman SA, Sheek-Hussein M, Bakir M, Trad O, Al-Hussani M, Uduman J, Sheikhs F. Pattern of varicella and associated complications in children in Unite Arab Emirates: 5-year descriptive study. East Mediterr Health J. 2009 Jul-Aug;15(4):800-6. PMID: 20187531.

118) Overwhelming sepsis presenting as sudden unexpected death
Sharief N, Khan K, Conlan P. Overwhelming sepsis presenting as sudden unexpected death. Arch Dis Child. 1993 Sep;69(3):381-2; discussion 382-3. doi: 10.1136/adc.69.3.381. PMID: 8215550; PMCID: PMC1062896.

119) Obturator internus muscle abscess in children: Report of seven cases and review
Viani RM, Bromberg K, Bradley JS. Obturator internus muscle abscess in children: report of seven cases and review. Clin Infect Dis. 1999 Jan;28(1):117-22. doi: 10.1086/515080. PMID: 10028081.

120) Numerous eruptive lesions of panniculitis associated with group A streptococcus bacteremia in an immunocompetent child

Pao W, Duncan KO, Bolognia JL, Carroll CB, Hotez PJ, Bessen DE. Numerous eruptive lesions of panniculitis associated with group A streptococcus bacteremia in an immunocompetent child. Clin Infect Dis. 1998 Sep;27(3):430-3. doi: 10.1086/514685. PMID: 9770136.

121) Neonatal pleural empyema with group A Streptococcus
Thaarup J, Ellermann-Eriksen S, Stjernholm J. Neonatal pleural empyema with group A Streptococcus. Acta Paediatr. 1997 Jul;86(7):769-71. doi: 10.1111/j.1651-2227.1997.tb08585.x. PMID: 9240890.

122) Neonatal Fournier's Gangrene: Avoiding Extensive Debridement.
De La Torre M, Solé C, Fanjul M, Berenguer B, Arriaga-Redondo M, de Tomás E, Lorca-García C. Neonatal Fournier's Gangrene: Avoiding Extensive Debridement. Pediatr Infect Dis J. 2021 Oct 1;40(10):e384-e387. doi: 10.1097/INF.0000000000003224. PMID: 34292272.

123) Necrotizing fasciitis with toxic shock syndrome in a child: a case report and review of literature.
Abass K, Saad H, Abd-Elsayed AA. Necrotizing fasciitis with toxic shock syndrome in a child: a case report and review of literature. Cases J. 2008 Oct 8;1(1):228. doi: 10.1186/1757-1626-1-228. PMID: 18842146; PMCID: PMC2577109.

124) Necrotizing fasciitis of the retroperitoneum: An unusual presentation of group a streptococcus infection
Devin B, McCarthy A, Mehran R, Auger C. Necrotizing fasciitis of the retroperitoneum: an unusual presentation of group A Streptococcus infection. Can J Surg. 1998 Apr;41(2):156-60. PMID: 9576000; PMCID: PMC3949830.

125) Multicenter study on invasive Streptococcus pyogenes infections in children in Argentina.
Cancellara AD, Melonari P, Firpo MV, Mónaco A, Ezcurra GC, Ruizf L, Aletti AM, Gregorio G, Gaiano A, Aird A, Bellone L, Calvari M, Torregrosa C, Morinigo S, Vozzan ML, Tonetto I, Flynn LP, Bidone NM, Russ C, Ellis A. Multicenter study on invasive Streptococcus pyogenes infections in children in Argentina. Arch Argent Pediatr. 2016 Jun 1;114(3):199-208. English, Spanish. doi: 10.5546/aap.2016.eng.199. Epub 2016 Apr 12. PMID: 27164331.

126) Localized meningoencephalitis and group A streptococcal bacteremia.
Levy EN, Griffith JA, Carvajal HF. Localized meningoencephalitis and group A streptococcal bacteremia. Clin Pediatr (Phila). 1992 Jul;31(7):438-41. doi: 10.1177/000992289203100712. PMID: 1617870.

127) Late onset neonatal sepsis caused by group A streptococcus.
Saito R, Kerr-Liddell R, Paul SP. Late onset neonatal sepsis caused by group A streptococcus. Br J Hosp Med (Lond). 2017 Mar 2;78(3):170-171. doi: 10.12968/hmed.2017.78.3.170. PMID: 28277774.

128) Invasive group A streptococcus infection of the scrotum and streptococcal toxic shock syndrome
Walker BR, Pribble CG, Cartwright PC. Invasive group A streptococcus infection of the scrotum and streptococcal toxic shock syndrome. Urology. 2000 Oct 1;56(4):669. doi: 10.1016/s0090-4295(00)00729-9. PMID: 11018632.

129) Invasive Group A Streptococcal Infections in Children: A Nationwide Survey in Finland.
Tapiainen T, Launonen S, Renko M, Saxen H, Salo E, Korppi M, Kainulainen L, Heiskanen-Kosma T, Lindholm L, Vuopio J, Huotari T, Rusanen J, Uhari M. Invasive Group A Streptococcal Infections in Children: A Nationwide Survey in Finland. Pediatr Infect Dis J. 2016 Feb;35(2):123-8. doi: 10.1097/INF.0000000000000945. PMID: 26440814.

130) Invasive group A streptococcal infection in children: Clinical manifestations and molecular characterization in a French pediatric tertiary care center

Henriet S, Kaguelidou F, Bidet P, Lorrot M, De Lauzanne A, Dauger S, Angoulvant F, Mercier JC, Alberti C, Bingen E, Faye A. Invasive group A streptococcal infection in children: clinical manifestations and molecular characterization in a French pediatric tertiary care center. Eur J Clin Microbiol Infect Dis. 2010 Mar;29(3):341-6. doi: 10.1007/s10096-009-0854-x. PMID: 20063027.

131) Intracranial hypertension secondary to sigmoid sinus compression by group A streptococcal epidural abscess
Ludemann JP, Poskitt K, Singhal A. Intracranial hypertension secondary to sigmoid sinus compression by group A streptococcal epidural abscess. J Laryngol Otol. 2010 Jan;124(1):93-5. doi: 10.1017/S0022215109990764. Epub 2009 Aug 3. PMID: 19646300.

132) Infectious Aneurysms Caused by Streptococcus Pyogenes in Children
Higuchi T, Ogawa E, Ikeyama T, Yasuda K, Murayama H, Hasegawa T, Ito K. Infectious Aneurysms Caused by Streptococcus Pyogenes in Children. Pediatr Infect Dis J. 2022 Oct 1;41(10):e442-e444. doi: 10.1097/INF.0000000000003651. Epub 2022 Jul 20. PMID: 35895891.

133) Incomplete limb ischemia as a complication in a pediatric patient with toxic-shock syndrome
Iliacus abscess in a child.
Wolfsberger, C. H., Pfurtscheller, K., Ulreich, R., Pocivalnik, M., Vasilyeva, A., & Schintler, M. V. (2019). Incomplete limb ischemia as a complication in a pediatric patient with toxic-shock syndrome. *Journal of Pediatric Surgery Case Reports*, *49*, 101282.

134) High-dose steroid and heparin: a novel therapy for cerebral vasculitis associated with presumed group A Streptococcus meningitis.
Hummel BA, Blackburn J, Pham-Huy A, Muir K. High-dose steroid and heparin: a novel therapy for cerebral vasculitis associated with presumed group A *Streptococcus* meningitis. BMJ Case Rep. 2021 Feb 9;14(2):e239618. doi: 10.1136/bcr-2020-239618. PMID: 33563670; PMCID: PMC7875284.

135) Group-A streptococcal infection in the newborn.
Coulter JB, Buchannon CR, Vellodi A, Hart CA, Sills JA. Group-A streptococcal infection in the newborn. Lancet. 1984 Aug 11;2(8398):355-6. doi: 10.1016/s0140-6736(84)92727-2. PMID: 6146901.

136) Group a streptococcus spinal epidural abscess during varicella.
Quach C, Tapiero B, Noya F. Group a streptococcus spinal epidural abscess during varicella. Pediatrics. 2002 Jan;109(1):E14. doi: 10.1542/peds.109.1.e14. PMID: 11773582.

137) Group a streptococcus mural endocarditis
Liu VC, Stevenson JG, Smith AL. Group A Streptococcus mural endocarditis. Pediatr Infect Dis J. 1992 Dec;11(12):1060-2. PMID: 1461701.

138) Group a streptococcal suppurative arthritis and osteomyelitis of the shoulder with brachial plexus palsy in a newborn
Dierig A, Ritz N, Tacke U, Heininger U. Group A Streptococcal Suppurative Arthritis and Osteomyelitis of the Shoulder With Brachial Plexus Palsy in a Newborn. Pediatr Infect Dis J. 2016 Oct;35(10):1151-3. doi: 10.1097/INF.0000000000001255. PMID: 27622687.

139) Group A Streptococcal Pericarditis in a Four-Month-Old Infant: Case report.
Al-Waili BR, Zacharias SK, Aslem E. Group A Streptococcal Pericarditis in a Four-Month-Old Infant: Case report. Sultan Qaboos Univ Med J. 2017 May;17(2):e241-e243. doi: 10.18295/squmj.2016.17.02.020. Epub 2017 Jun 20. PMID: 28690902; PMCID: PMC5488831.

140) Group A streptococcal panniculitis

Villaseñor-Park J, Davis A, Singh S, Zuckerbraun N, Green M, Gehris R. Group A streptococcal panniculitis. Pediatr Dermatol. 2014 Mar-Apr;31(2):256-8. doi: 10.1111/j.1525-1470.2012.01816.x. Epub 2012 Aug 1. PMID: 22856529.

141) Group A streptococcal meningitis and brain abscess.
Jagdis F. Group A streptococcal meningitis and brain abscess. Pediatr Infect Dis J. 1988 Dec;7(12):885-6. PMID: 3062565.

142) Group A streptococcal brain abscess in children: two case reports and a review of the literature.
Hazan G, Kristal E, Gideon M, Tzudikov V, Cavari Y, Fruchtman Y, Ben-Shimol S, Leibovitz E, Lazar I, Melamed R. Group A streptococcal brain abscess in children: two case reports and a review of the literature. Infect Dis (Lond). 2018 Feb;50(2):145-149. doi: 10.1080/23744235.2017.1374551. Epub 2017 Sep 12. PMID: 28895764.

143) Group a β-hemolytic streptococcal meningitis associated with uncomplicated varicella
Walsh M, Chodock R, Quinn C, Peglow S. Group A beta-hemolytic streptococcal meningitis associated with uncomplicated varicella. Am J Emerg Med. 1994 Sep;12(5):602-3. doi: 10.1016/0735-6757(94)90280-1. PMID: 8060414.

144) Fulminant Streptococcus pyogenes infection.
Conner WT. Fulminant Streptococcus pyogenes infection. Br Med J (Clin Res Ed). 1981 Feb 21;282(6264):651. doi: 10.1136/bmj.282.6264.651. PMID: 6781605; PMCID: PMC1504440.

145) Fatal streptococcal toxic shock syndrome in a child with varicella and necrotizing fasciitis of the face
Minodier P, Chaumoitre K, Vialet R, Imbert G, Bidet P. Fatal streptococcal toxic shock syndrome in a child with varicella and necrotizing fasciitis of the face. Eur J Emerg Med. 2008 Aug;15(4):231-3. doi: 10.1097/MEJ.0b013e3282f08d3d. PMID: 19078822.

146) Fatal group a streptococcal meningitis and toxic shock-like syndrome: Case report
Jevon GP, Dunne WM Jr, Hawkins HK, Armstrong DL, Musser JM. Fatal group A streptococcal meningitis and toxic shock-like syndrome: case report. Clin Infect Dis. 1994 Jan;18(1):91-3. doi: 10.1093/clinids/18.1.91. PMID: 8054439.

147) Endocarditis caused by group A beta-hemolytic Streptococcus in an infant: case report and review.
Winterbotham A, Riley S, Kavanaugh-McHugh A, Dermody TS. Endocarditis caused by group A beta-hemolytic Streptococcus in an infant: case report and review. Clin Infect Dis. 1999 Jul;29(1):196-8. doi: 10.1086/520153. PMID: 10433586.

148) Endocarditis attributable to group A beta-hemolytic streptococcus after uncomplicated varicella in a vaccinated child.
Laskey AL, Johnson TR, Dagartzikas MI, Tobias JD. Endocarditis attributable to group A beta-hemolytic streptococcus after uncomplicated varicella in a vaccinated child. Pediatrics. 2000 Sep;106(3):E40. doi: 10.1542/peds.106.3.e40. PMID: 10969124.

149) Diagnosis and management of deep neck infections in children: The experience of an Italian paediatric centre
Raffaldi I, Le Serre D, Garazzino S, Scolfaro C, Bertaina C, Mignone F, Peradotto F, Tavormina P, Tovo PA. Diagnosis and management of deep neck infections in children: the experience of an Italian paediatric centre. J Infect Chemother. 2015 Feb;21(2):110-3. doi: 10.1016/j.jiac.2014.10.011. Epub 2014 Nov 20. PMID: 25456894.

150) Complications associated with severe invasive streptococcal syndrome.

Montgomery VL, Bratcher D. Complications associated with severe invasive streptococcal syndrome. J Pediatr. 1996 Oct;129(4):602-4. doi: 10.1016/s0022-3476(96)70127-0. PMID: 8859269.

151) Compartment Syndrome Secondary to Group A Streptococcus Infection in the Presence of a Congenital Hemangioma.
Kracoff SL. Compartment Syndrome Secondary to Group A Streptococcus Infection in the Presence of a Congenital Hemangioma. Pediatr Emerg Care. 2019 Apr;35(4):290-292. doi: 10.1097/PEC.0000000000000952. PMID: 27798538.

152) Clinical Manifestations and Bacterial Genomic Analysis of Group A Streptococcus Strains That Cause Pediatric Toxic Shock Syndrome.
Deniskin R, Shah B, Muñoz FM, Flores AR. Clinical Manifestations and Bacterial Genomic Analysis of Group A Streptococcus Strains That Cause Pediatric Toxic Shock Syndrome. J Pediatric Infect Dis Soc. 2019 Jul 1;8(3):265-268. doi: 10.1093/jpids/piy069. PMID: 30085250; PMCID: PMC6601382.

153) An unusual cutaneous manifestation of group A streptococcal bacteremia.
Ortbals DW, Glew RH, Santa Cruz DJ. An unusual cutaneous manifestation of group A streptococcal bacteremia. South Med J. 1978 Nov;71(11):1421-3. doi: 10.1097/00007611-197811000-00031. PMID: 362541.

154) Acute bacterial meningitis caused by group a streptococcus in Varicella: a case report
Calik, Mustaf. "Acute bacterial meningitis caused by group a streptococcus in Varicella: a case report." American Journal of Case Reports 9 (2008): 170-172.
APA

155) A family cluster of streptococcal toxic shock syndrome in children: clinical implication and epidemiological investigation.
Huang YC, Hsueh PR, Lin TY, Yan DC, Hsia SH. A family cluster of streptococcal toxic shock syndrome in children: clinical implication and epidemiological investigation. Pediatrics. 2001 May;107(5):1181-3. doi: 10.1542/peds.107.5.1181. PMID: 11331706.

156) A Cluster of Pediatric Invasive Group A Streptococcus Disease in Melbourne, Australia, Coinciding with a High-Burden Influenza Season
Wong, Nicole X., et al. "A cluster of pediatric invasive group A streptococcus disease in Melbourne, Australia, coinciding with a high-burden influenza season." *Journal of Pediatric Infectious Diseases* 14.04 (2019): 213-218.

157) A 31-day-old infant with late-onset sepsis and complicated pneumonia due to streptococcus pyogenes
Bekkam G.J.; Dumpa V.; Tolan Jr. R.W. A 31-day-old infant with late-onset sepsis and complicated pneumonia due to streptococcus pyogenes. Infectious Diseases in Clinical Practice. 2014.

158) Varicella complicated by cellulitis and deep vein thrombosis
Oliveira GN, Basso S, Sevivas T, Neves N. Varicella complicated by cellulitis and deep vein thrombosis. BMJ Case Rep. 2017 Sep 7;2017:bcr2017221499. doi: 10.1136/bcr-2017-221499. PMID: 28882940; PMCID: PMC5589027.

159) Uncommon clinical presentation of a common bug: Group A Streptococcus meningitis.
Lee J, Blackburn J, Pham-Huy A. Uncommon clinical presentation of a common bug: Group A *Streptococcus* meningitis. Paediatr Child Health. 2020 May 28;26(3):e129-e131. doi: 10.1093/pch/pxaa065. PMID: 33936341; PMCID: PMC8077209.

SUPPLEMENTARY MATERIAL: LIST OF STUDIES INCLUDED IN THE SCOPING REVIEW

160) Trivial trauma, lethal outcome: Streptococcal toxic shock syndrome presenting to the ED
Lin JN, Chang LL, Lai CH, Lin HH, Chen YH. Trivial trauma, lethal outcome: streptococcal toxic shock syndrome presenting to the ED. Am J Emerg Med. 2013 Aug;31(8):1293.e1-3. doi: 10.1016/j.ajem.2013.04.011. Epub 2013 May 21. PMID: 23702056.

161) Toxic shock syndrome in Australian children.
Chen KY, Cheung M, Burgner DP, Curtis N. Toxic shock syndrome in Australian children. Arch Dis Child. 2016 Aug;101(8):736-40. doi: 10.1136/archdischild-2015-310121. Epub 2016 Apr 26. PMID: 27117838.

162) Subgaleal abscess: An unusual presentation
Wiley JF 2nd, Sugarman JM, Bell LM. Subgaleal abscess: an unusual presentation. Ann Emerg Med. 1989 Jul;18(7):785-7. doi: 10.1016/s0196-0644(89)80021-6. PMID: 2660643.

163) Streptococcus pyogenes pyomyositis.
Zervas SJ, Zemel LS, Romness MJ, Kaplan EL, Salazar JC. Streptococcus pyogenes pyomyositis. Pediatr Infect Dis J. 2002 Feb;21(2):166-8. doi: 10.1097/00006454-200202000-00017. PMID: 11840087.

164) Streptococcus pyogenes meningitis in children: Report of two cases and literature review
Arnoni MV, Berezin EN, Sáfadi MA, Almeida FJ, Lopes CR. Streptococcus pyogenes meningitis in children: report of two cases and literature review. Braz J Infect Dis. 2007 Jun;11(3):375-7. doi: 10.1590/s1413-86702007000300015. PMID: 17684643.

165) Streptococcus pyogenes meningitis complicating varicella in a 3-month-old child
Brandt CM, Kitz R, Lütticken R, Brade V. Streptococcus pyogenes meningitis complicating varicella in a 3-month-old child. Scand J Infect Dis. 2003;35(11-12):876-8. doi: 10.1080/00365540310016844. PMID: 14723366.

166) Streptococcus pyogenes meningitis.
Mathur P, Arora NK, Kapil A, Das BK. Streptococcus pyogenes meningitis. Indian J Pediatr. 2004 May;71(5):423-6. doi: 10.1007/BF02725632. PMID: 15163872.

167) Streptococcus pyogenes Endocarditis Associated With Varicella-Case Report and Review of the Literature.
Savoia P, Heininger U, Buettcher M. *Streptococcus pyogenes* Endocarditis Associated With Varicella-Case Report and Review of the Literature. Front Pediatr. 2019 Dec 4;7:500. doi: 10.3389/fped.2019.00500. PMID: 31867295; PMCID: PMC6904297.

168) Streptococcal toxic shock–like syndrome as a complication of varicella
Bradley JS, Schlievert PM, Sample TG Jr. Streptococcal toxic shock-like syndrome as a complication of varicella. Pediatr Infect Dis J. 1991 Jan;10(1):77-9. doi: 10.1097/00006454-199101000-00017. PMID: 2003062.

169) Streptococcal toxic shock syndrome presenting as septic knee arthritis in a 5-year-old child
Alwattar BJ, Strongwater A, Sala DA. Streptococcal toxic shock syndrome presenting as septic knee arthritis in a 5-year-old child. J Pediatr Orthop. 2008 Jan-Feb;28(1):124-7. doi: 10.1097/bpo.0b013e31815b4dfd. PMID: 18157057.

170) Streptococcal toxic shock syndrome manifesting as peritonitis in a child
Liang TC, Lu CY, Lu FL, Lee PI, Huang LM. Streptococcal toxic shock syndrome manifesting as peritonitis in a child. J Formos Med Assoc. 2002 Jul;101(7):509-13. PMID: 12353345.

171) Streptococcal Toxic Shock Syndrome in the Emergency Department

SUPPLEMENTARY MATERIAL: LIST OF STUDIES INCLUDED IN THE SCOPING REVIEW

Guitart-Pardellans, Carmina, et al. "Streptococcal Toxic Shock Syndrome in the Emergency Department." Journal of Pediatric Infectious Diseases 15.02 (2020): 091-094.

172) Streptococcal toxic shock syndrome in children without skin and soft tissue infection: Report of four cases
Chiang MC, Jaing TH, Wu CT, Hsia SH, Chiu CH. Streptococcal toxic shock syndrome in children without skin and soft tissue infection: report of four cases. Acta Paediatr. 2005 Jun;94(6):763-5. doi: 10.1111/j.1651-2227.2005.tb01979.x. PMID: 16188783.

173) Streptococcal toxic shock syndrome due to noninvasive pharyngitis
Chapnick EK, Gradon JD, Lutwick LI, Kim J, Levi M, Kim MH, Schlievert PM. Streptococcal toxic shock syndrome due to noninvasive pharyngitis. Clin Infect Dis. 1992 May;14(5):1074-7. doi: 10.1093/clinids/14.5.1074. PMID: 1600009.

174) Streptococcal toxic shock in three alabama children
Ware JC, Eich WF, Ruben EB, Malone JA, Schlievert PM, Gray BM. Streptococcal toxic shock in three Alabama children. Pediatr Infect Dis J. 1993 Sep;12(9):765-9. doi: 10.1097/00006454-199309000-00013. PMID: 8414805.

175) Streptococcal pyomyositis of the psoas: case reports and review.
Kern L, Rassbach C, Ottolini M. Streptococcal pyomyositis of the psoas: case reports and review. Pediatr Emerg Care. 2006 Apr;22(4):250-3. doi: 10.1097/01.pec.0000210177.48386.e7. PMID: 16651916.

176) Streptococcal Preseptal Cellulitis Complicated by the Toxic Streptococcus Syndrome
Ingraham HJ, Ryan ME, Burns JT, Shuhart D, Tenedios G, Malone W, Bitterly T, Youn B, Huffard R. Streptococcal preseptal cellulitis complicated by the toxic Streptococcus syndrome. Ophthalmology. 1995 Aug;102(8):1223-6. doi: 10.1016/s0161-6420(95)30886-x. PMID: 9097751.

177) Streptococcal pancreatitis and toxic shock syndrome in a 2-month-old infant
Adams D, Fenton SJ, Nichol PF. Streptococcal pancreatitis and toxic shock syndrome in a 2-month-old infant. J Pediatr Surg. 2007 Jan;42(1):261-3. doi: 10.1016/j.jpedsurg.2006.09.026. PMID: 17208579.

178) Streptococcal necrotizing fasciitis with toxic shock syndrome following cervical adenitis.
Sakran W, Mazzawi S, Merzel Y, Colodner R. Streptococcal necrotizing fasciitis with toxic shock syndrome following cervical adenitis. Int J Pediatr Otorhinolaryngol. 2004 Sep;68(9):1209-13. doi: 10.1016/j.ijporl.2004.04.019. PMID: 15302155.

179) Spinal epidural abscess from group A Streptococcus after varicella infection: a case report and review of the literature
Cossu G, Farhane MA, Daniel RT, Messerer M. Spinal epidural abscess from group A Streptococcus after varicella infection: a case report and review of the literature. Childs Nerv Syst. 2014 Dec;30(12):2129-33. doi: 10.1007/s00381-014-2479-3. Epub 2014 Jul 8. PMID: 25001474.

180) Severe neonatal group A streptococcal disease.
Verboon-Maciolek MA, Krediet TG, van Ertbruggen I, Gerards LJ, Fleer A. Severe neonatal group A streptococcal disease. Eur J Pediatr. 2000 Jun;159(6):450-2. doi: 10.1007/s004310051305. PMID: 10867852.

181) Serious suppurative group a streptococcal infections in previously well children
Harnden A, Lennon D. Serious suppurative group A streptococcal infections in previously well children. Pediatr Infect Dis J. 1988 Oct;7(10):714-8. doi: 10.1097/00006454-198810000-00010. PMID: 3054779.

182) Serious group A streptococcal diseases in children.

Burech DL, Koranyi KI, Haynes RE. Serious group A streptococcal diseases in children. J Pediatr. 1976 Jun;88(6):972-4. doi: 10.1016/s0022-3476(76)81052-9. PMID: 775044.

183) Serious Group A β-Hemolytic Streptococcal Infections Complicating Varicella
Cowan MR, Primm PA, Scott SM, Abramo TJ, Wiebe RA. Serious group A beta-hemolytic streptococcal infections complicating varicella. Ann Emerg Med. 1994 Apr;23(4):818-22. doi: 10.1016/s0196-0644(94)70320-5. PMID: 8161053.

184) Sepsis and Pleural Empyema Caused by Streptococcus pyogenes after Influenza A Virus Infection.
Ochi F, Tauchi H, Jogamoto T, Miura H, Moritani T, Nagai K, Ishii E. Sepsis and Pleural Empyema Caused by *Streptococcus pyogenes*after Influenza A Virus Infection. Case Rep Pediatr. 2018 Sep 23;2018:4509847. doi: 10.1155/2018/4509847. PMID: 30345134; PMCID: PMC6174774.

185) Risk factors for pediatric invasive group A streptococcal disease.
Factor SH, Levine OS, Harrison LH, Farley MM, McGeer A, Skoff T, Wright C, Schwartz B, Schuchat A. Risk factors for pediatric invasive group A streptococcal disease. Emerg Infect Dis. 2005 Jul;11(7):1062-6. doi: 10.3201/eid1107.040900. PMID: 16022781; PMCID: PMC3371775.

186) Retiform Purpura as a Sign of Necrotizing Cellulitis in an Immunocompetent Boy.
Aboul-Fettouh N, Nguyen KD, Dominguez AR. Retiform Purpura as a Sign of Necrotizing Cellulitis in an Immunocompetent Boy. Pediatr Emerg Care. 2020 Nov;36(11):e646-e648. doi: 10.1097/PEC.0000000000002251. PMID: 32970024.

187) Recurrent fatal necrotizing fasciitis due to Streptococcus pyogenes in a child with hereditary sensory and automic neuropathy type IV
Kuzdan C, Soysal A, Altinkanat G, Aksu B, Söyletir G, Bakir M. Recurrent fatal necrotizing fasciitis due to Streptococcus pyogenes in a child with hereditary sensory and automic neuropathy type IV. Jpn J Infect Dis. 2011;64(2):147-9. PMID: 21519130.

188) Rapid development of brain abscess caused by streptococcus pyogenes following penetrating skull injury via the ethmoidal sinus and lamina cribrosa
Gulsen S, Aydin G, Cömert S, Altinors N. Rapid Development of Brain Abscess Caused by Streptococcus Pyogenes Following Penetrating Skull Injury via the Ethmoidal Sinus and Lamina Cribrosa. J Korean Neurosurg Soc. 2010 Jul;48(1):73-8. doi: 10.3340/jkns.2010.48.1.73. Epub 2010 Jul 31. PMID: 20717517; PMCID: PMC2916153.

189) Prospective Surveillance of Pediatric Invasive Group A Streptococcus Infection.
Ching NS, Crawford N, McMinn A, Baker C, Azzopardi K, Brownlee K, Lee D, Gibson M, Smeesters P, Gonis G, Ojaimi S, Buttery J, Steer AC. Prospective Surveillance of Pediatric Invasive Group A Streptococcus Infection. J Pediatric Infect Dis Soc. 2019 Mar 28;8(1):46-52. doi: 10.1093/jpids/pix099. PMID: 29309631.

190) Prolonged morbidity in children with group A beta-hemolytic streptococcal pneumonia.
Trujillo M, McCracken GH Jr. Prolonged morbidity in children with group A beta-hemolytic streptococcal pneumonia. Pediatr Infect Dis J. 1994 May;13(5):411-2. PMID: 8072825.

191) Primary peritonitis due to group A Streptococcus in a previously healthy pediatric patient
Holden R, Wilmer A, Kollman T. Primary peritonitis due to group A Streptococcus in a previously healthy pediatric patient. Can J Infect Dis Med Microbiol. 2012 Fall;23(3):e69-70. doi: 10.1155/2012/105850. PMID: 23997789; PMCID: PMC3476566.

192) Primary group a streptococcal peritonitis in a previously healthy child

Demitrack J. Primary group A streptococcal peritonitis in a previously healthy child. Pediatr Infect Dis J. 2012 May;31(5):542-3. doi: 10.1097/INF.0b013e31824f1b0d. PMID: 22510999.

193) Post-varicella epiglottitis and necrotizing fasciitis.
Slack CL, Allen GC, Morrison JE, Garren KC, Roback MG. Post-varicella epiglottitis and necrotizing fasciitis. Pediatrics. 2000 Jan;105(1):e13. doi: 10.1542/peds.105.1.e13. PMID: 10617750.

194) Posterior mediastinal abscess caused by invasive group A Streptococcus infection
Conway JH, Nyquist AC, Goldson E. Posterior mediastinal abscess caused by invasive group A Streptococcus infection. Pediatr Infect Dis J. 1996 Jun;15(6):547-9. doi: 10.1097/00006454-199606000-00018. PMID: 8783358.

195) Paediatric Minor facial trauma resulting in invasive Group A streptococcal orofacial infections: two case reports
Maarouf, Marwa, et al. "Paediatric Minor facial trauma resulting in invasive Group A streptococcal orofacial infections: two case reports." *Oral Surgery* 12.3 (2019): 268-271.

196) Paediatric case of group A streptococcal pharyngitis, arthritis and osteomyelitis associated with dental neglect.
Hiraoka T, Chujo T, Tsuge M, Kondo Y. Paediatric case of group A streptococcal pharyngitis, arthritis and osteomyelitis associated with dental neglect. BMJ Case Rep. 2021 Jan 18;14(1):e239196. doi: 10.1136/bcr-2020-239196. PMID: 33462043; PMCID: PMC7816913.

197) Otherwise Healthy 15-Month-Old with Rapid-Onset Soft Tissue Swelling
Anderson JJ, Avendano P, Kaila R. Otherwise Healthy 15-Month-Old With Rapid-Onset Soft Tissue Swelling. Pediatr Emerg Care. 2019 Aug;35(8):e138-e140. doi: 10.1097/PEC.0000000000001888. PMID: 31373950.

198) Neonatal septicemia caused by group A beta–hemolytic streptococcus
Wilschanski M, Faber J, Abramov A, Isacsohn M, Erlichman M. Neonatal septicemia caused by group A beta-hemolytic Streptococcus. Pediatr Infect Dis J. 1989 Aug;8(8):536-7. doi: 10.1097/00006454-198908000-00015. PMID: 2671908.

199) Neonatal septicaemia due to group A beta-haemolytic streptococcus.
Cartwright RY. Neonatal septicaemia due to group A beta-haemolytic streptococcus. Br Med J. 1977 Jan 15;1(6054):146-7. doi: 10.1136/bmj.1.6054.146. PMID: 318899; PMCID: PMC1603893.

200) Neonatal pleural empyema caused by emm type 6 group A streptococcus.
Nohara F, Nagaya K, Asai H, Tsuchida E, Okamoto T, Hayashi T, Sakata H, Terao Y, Azuma H. Neonatal pleural empyema caused by emm type 6 group A streptococcus. Pediatr Int. 2013 Aug;55(4):519-21. doi: 10.1111/ped.12061. PMID: 23910803.

201) Neonatal invasive group A streptococcal disease: case report and review of the literature.
Miyairi I, Berlingieri D, Protic J, Belko J. Neonatal invasive group A streptococcal disease: case report and review of the literature. Pediatr Infect Dis J. 2004 Feb;23(2):161-5. doi: 10.1097/01.inf.0000109887.40636.07. PMID: 14872185.

202)Neonatal group A streptococcal meningitis: a case report and review of the literature.
Lardhi AA. Neonatal group A streptococcal meningitis: a case report and review of the literature. Cases J. 2008 Aug 18;1(1):108. doi: 10.1186/1757-1626-1-108. PMID: 18710558; PMCID: PMC2531175.

203) Neonatal cellulitis and sepsis caused by group A streptococcus

Martic J, Mijac V, Jankovic B, Sekulovic LK, Vasiljevic Z, Vuksanovic J. Neonatal cellulitis and sepsis caused by group A streptococcus. Pediatr Dermatol. 2010 Sep-Oct;27(5):528-30. doi: 10.1111/j.1525-1470.2010.01262.x. Epub 2010 Aug 27. PMID: 20807361.

204) Necrotizing fasciitis of the scalp in a newborn
Davey C, Moore AM. Necrotizing fasciitis of the scalp in a newborn. Obstet Gynecol. 2006 Feb;107(2 Pt 2):461-3. doi: 10.1097/01.AOG.0000164094.02571.77. PMID: 16449149.

205) Necrotizing fasciitis in children due to minor lesions
Pfeifle, Viktoria A., et al. "Necrotizing fasciitis in children due to minor lesions." *Journal of pediatric surgery case reports* 25 (2017): 52-55.

206) Necrotizing fasciitis in children: diagnostic and therapeutic aspects.
Bingöl-Koloğlu M, Yildiz RV, Alper B, Yağmurlu A, Ciftçi E, Gökçora IH, Ince E, Emiroğlu M, Dindar H. Necrotizing fasciitis in children: diagnostic and therapeutic aspects. J Pediatr Surg. 2007 Nov;42(11):1892-7. doi: 10.1016/j.jpedsurg.2007.07.018. PMID: 18022442.

207) Necrotizing fasciitis and streptococcal toxic shock syndrome secondary to varicella in a healthy child
Kwak BO, Lee MJ, Park HW, Song MK, Chung S, Kim KS. Necrotizing fasciitis and streptococcal toxic shock syndrome secondary to varicella in a healthy child. Korean J Pediatr. 2014 Dec;57(12):538-41. doi: 10.3345/kjp.2014.57.12.538. Epub 2014 Dec 31. PMID: 25653688; PMCID: PMC4316598.

208) Necrotising fasciitis complicating varicella
de Benedictis FM, Osimani P. Necrotising fasciitis complicating varicella. BMJ Case Rep. 2009;2009:bcr2008141994. doi: 10.1136/bcr.2008.141994. Epub 2009 Jan 8. PMID: 21687323; PMCID: PMC3034804.

209) Group A streptococcal brain abscess.
Khan MA, Viagappan GM, Andrews J. Group A streptococcal brain abscess. Scand J Infect Dis. 2001;33(2):159. doi: 10.1080/003655401750065607. PMID: 11233856.
